# Supplementary figures and images for: IntroUNET: Identifying introgressed alleles via semantic segmentation
Source: PLoS Genet. 2024 Feb 20;20(2):e1010657. doi: 10.1371/journal.pgen.1010657 (PMC10906877; doi:10.1371/journal.pgen.1010657)

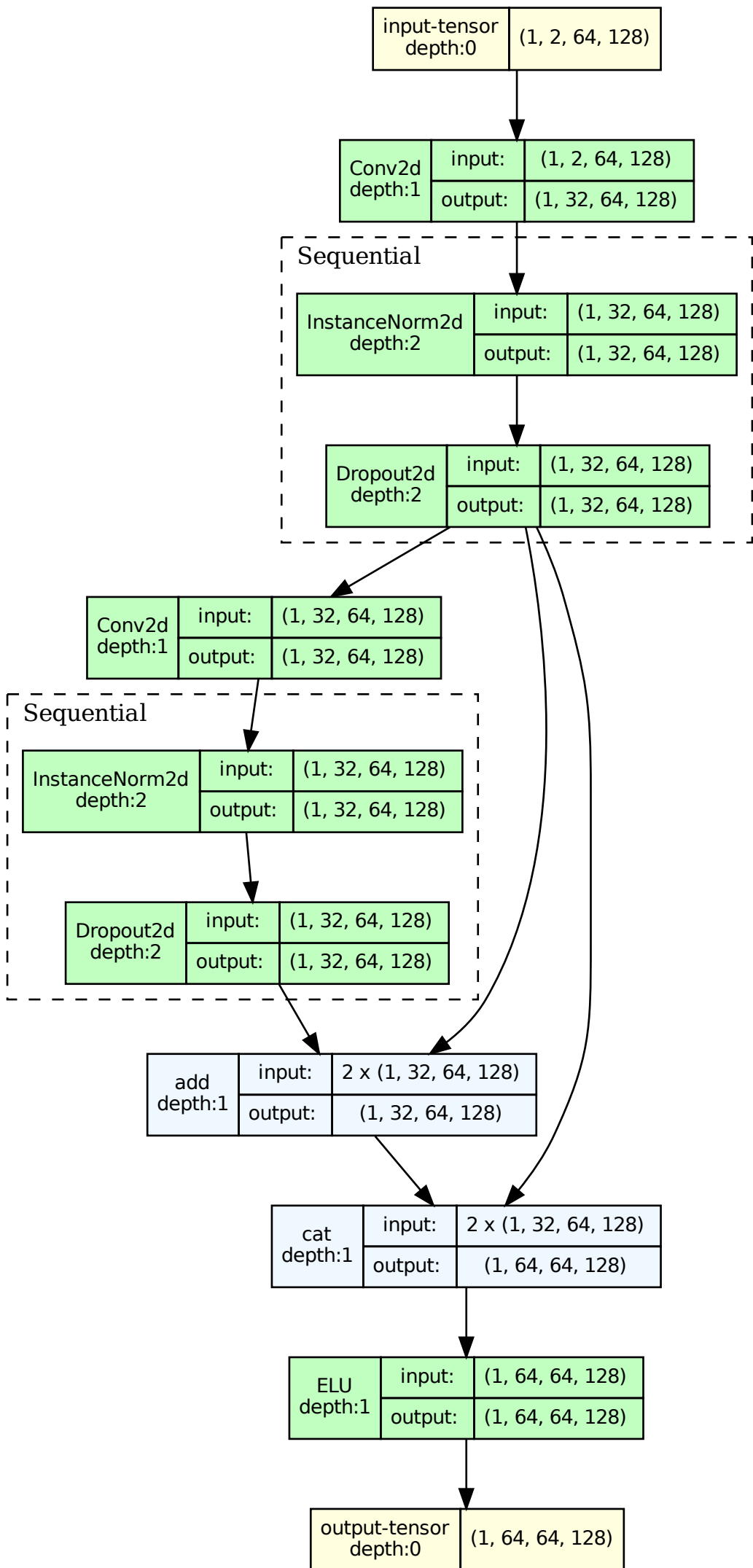

Supplement: S1 Fig — (PDF) [file pgen.1010657.s001.pdf]

Training/validation loss vs. strength of label smoothing

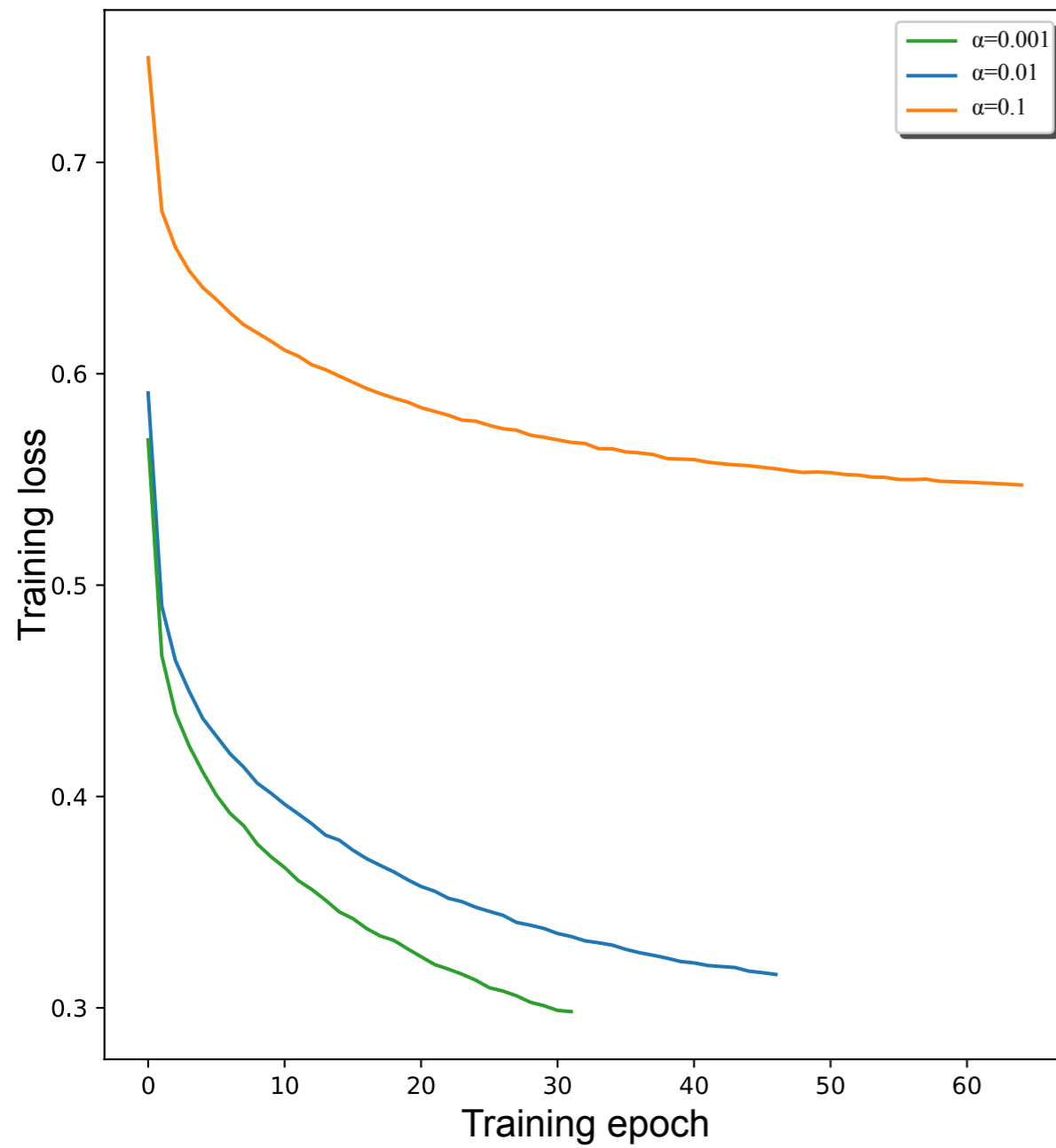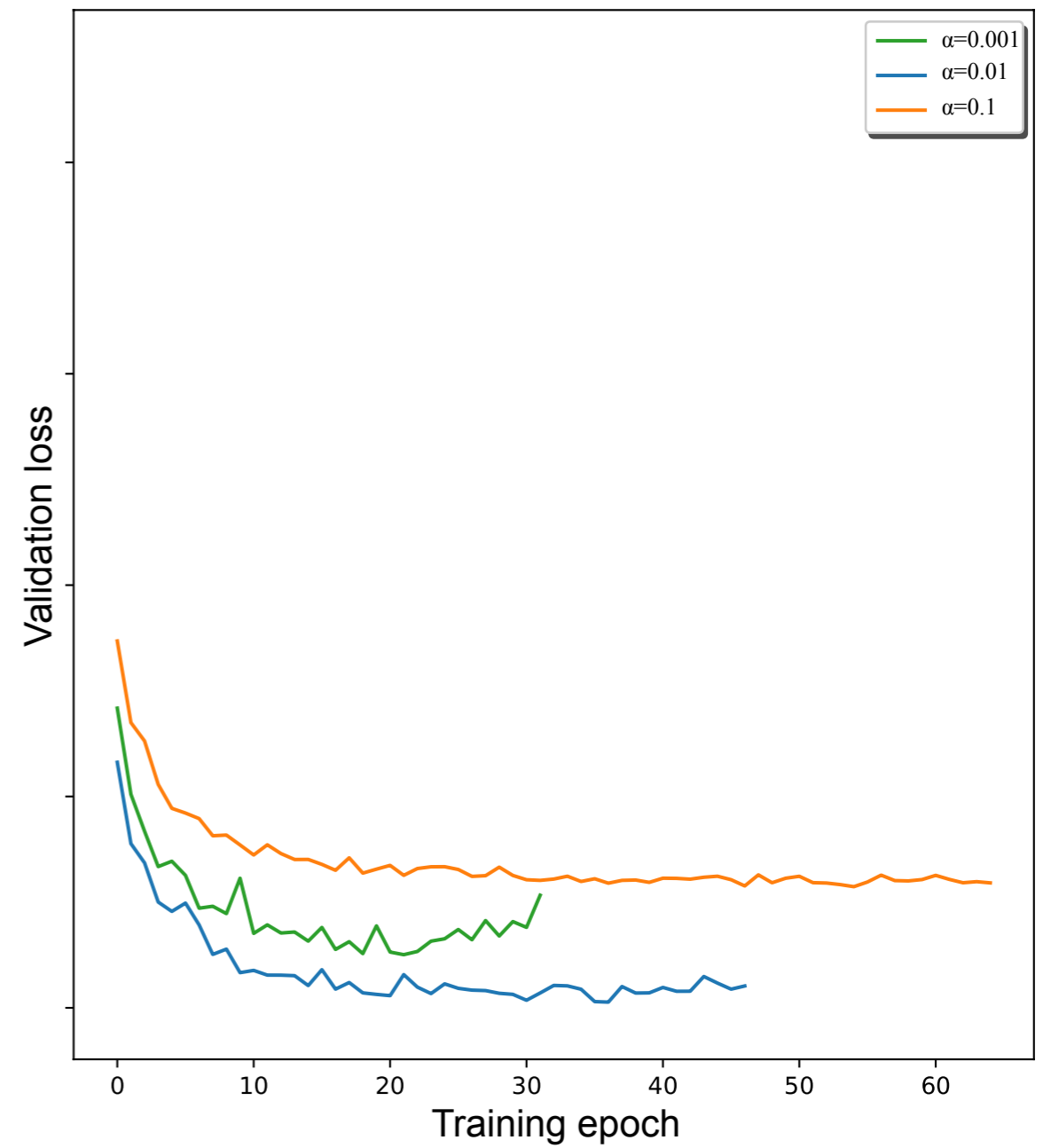

Supplement: S2 Fig — All tests were calculated on simulated examples of the simple bidirectional scenario described in the Methods. Note that for alpha = 0.1 training loss is higher than validation loss. This is because label smoothing is only applied during training, and smoothing increases loss by adding noise to the target y values. (PDF) [file pgen.1010657.s002.pdf]

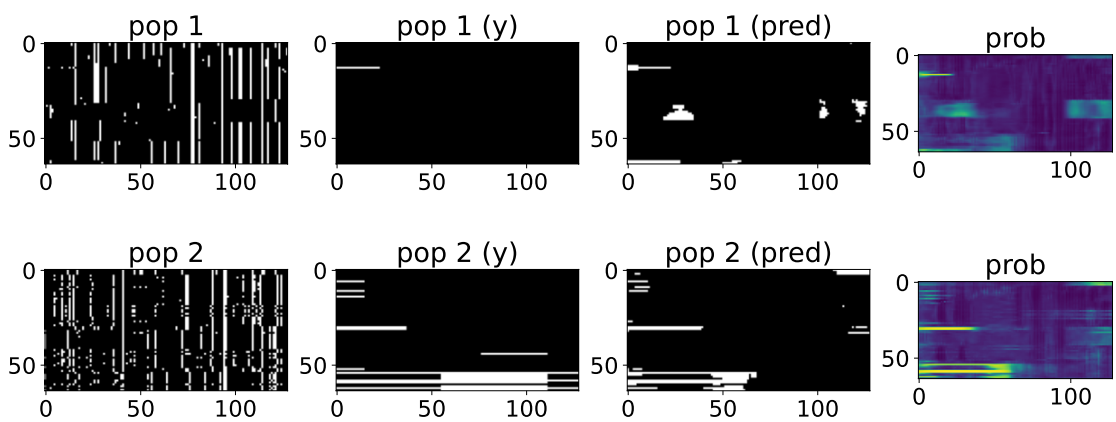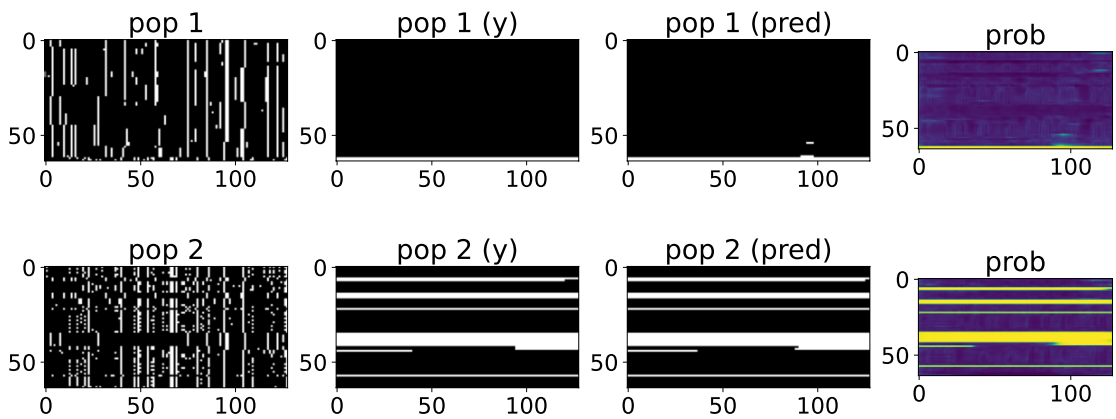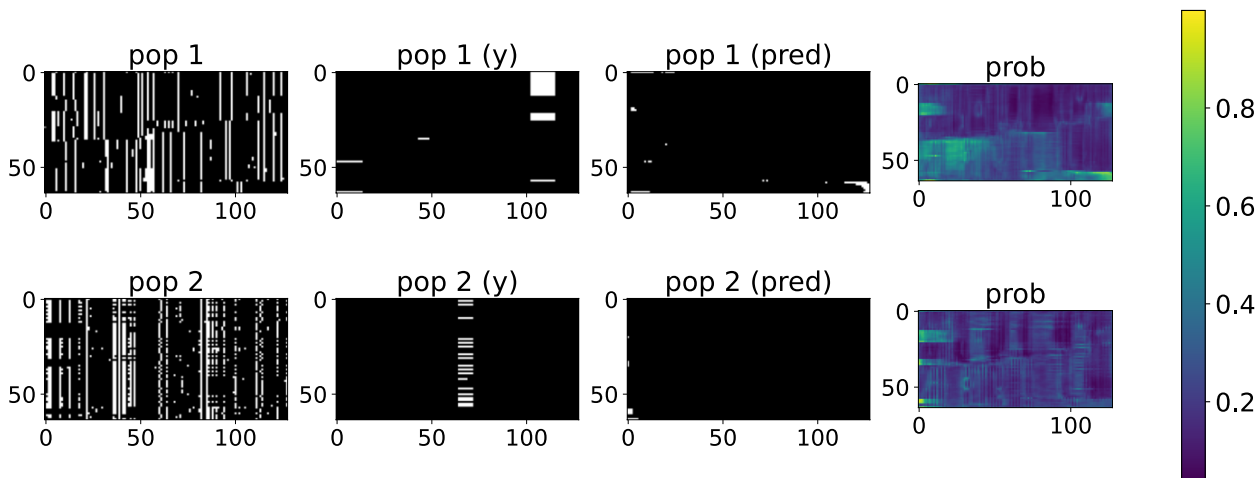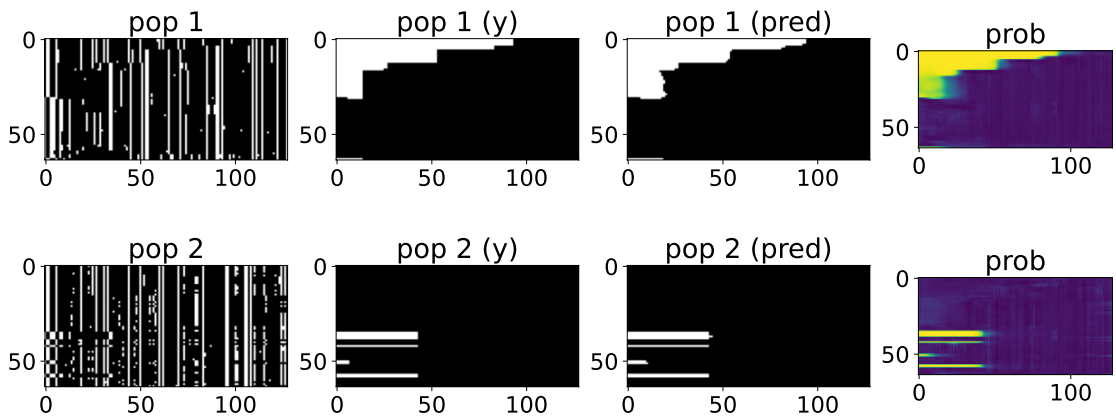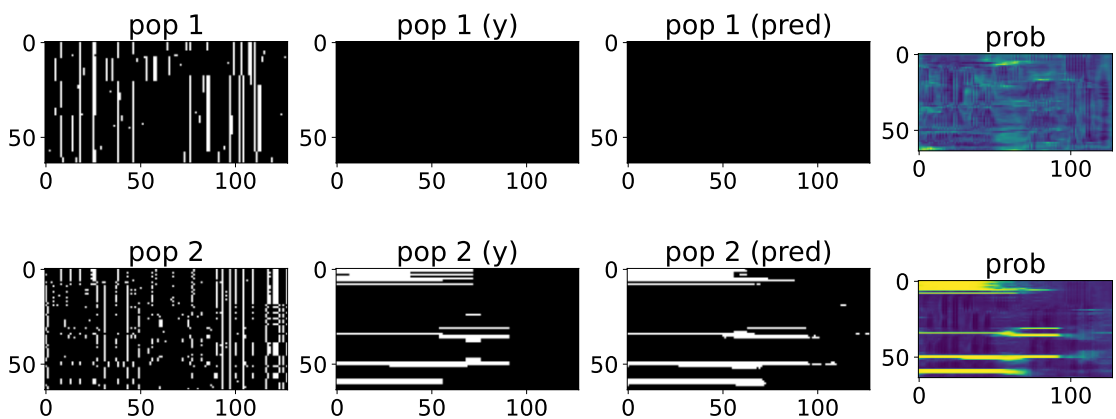

Supplement: S3 Fig — Each example shows the input alignments for the two populations (labeled “pop 1” and “pop 2” respectively), the true introgressed alleles for these two populations (labeled “pop 1 (y)” and “pop 2 (y)” respectively), the introgressed alleles inferred by IntroUNET (“pop 1 (pred)” and “pop 2 (pred)”), and IntroUNET’s inferred introgression probabilities (labelled “prob”, and scaled according to the color bar shown with the third example). Alignments and introgressed histories, true and predicted, are shown in the same format as in Fig 1. (PDF) [file pgen.1010657.s003.pdf]

Loss vs. sample size

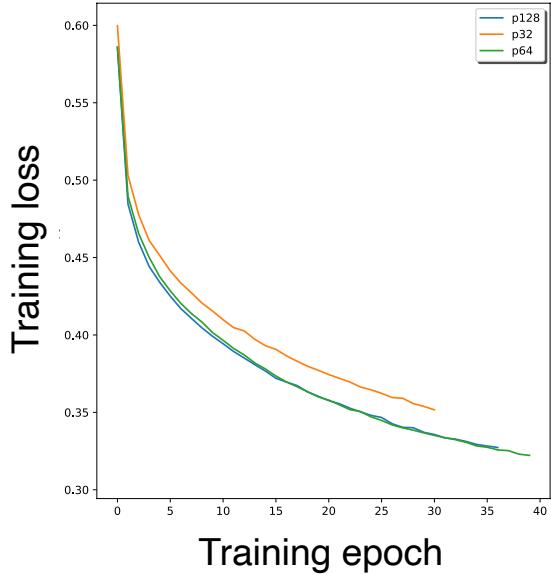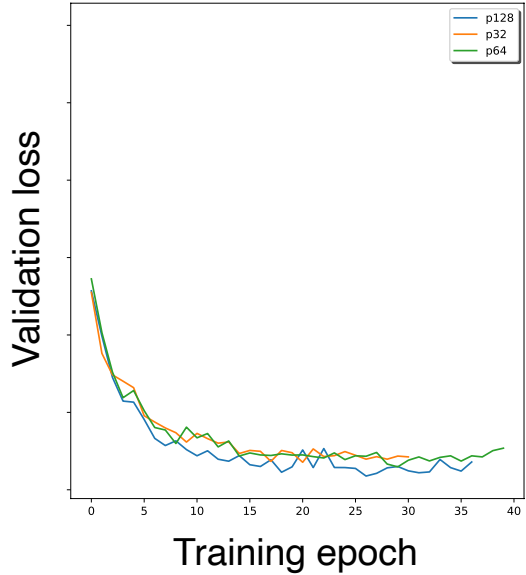

Loss vs. number of training examples

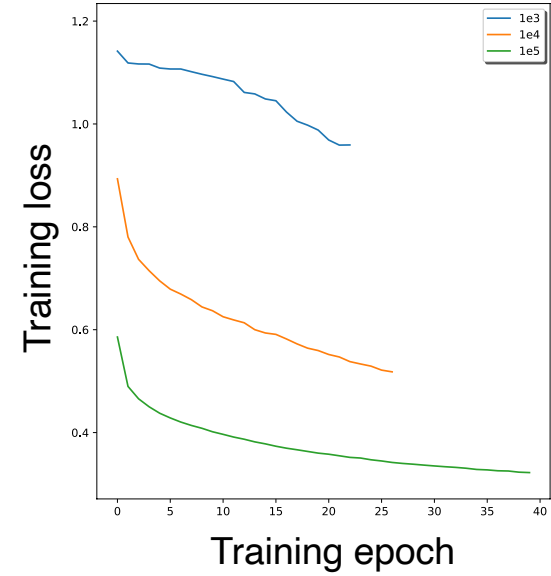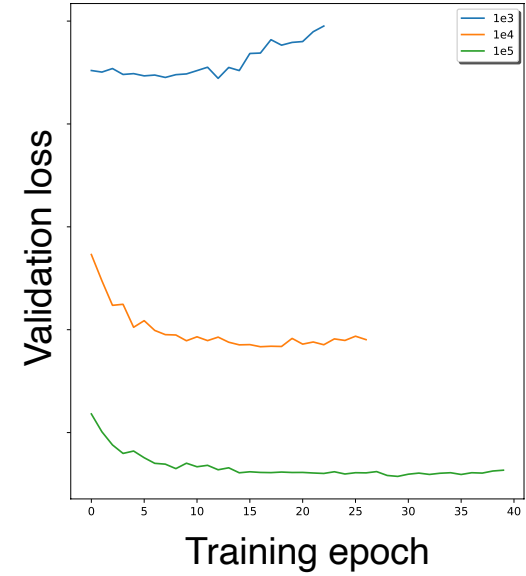

Loss vs. alignment size

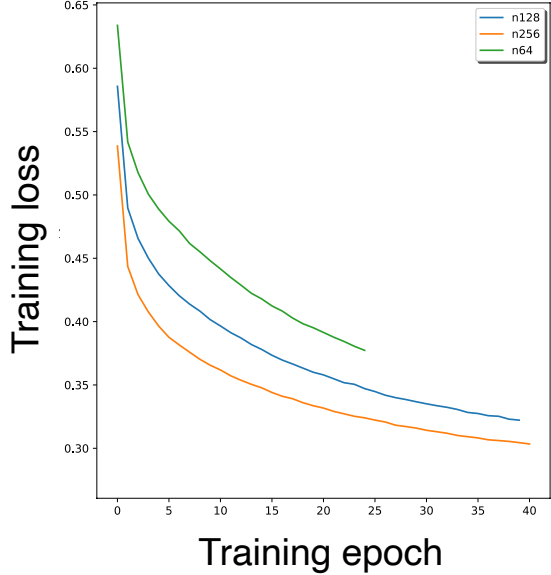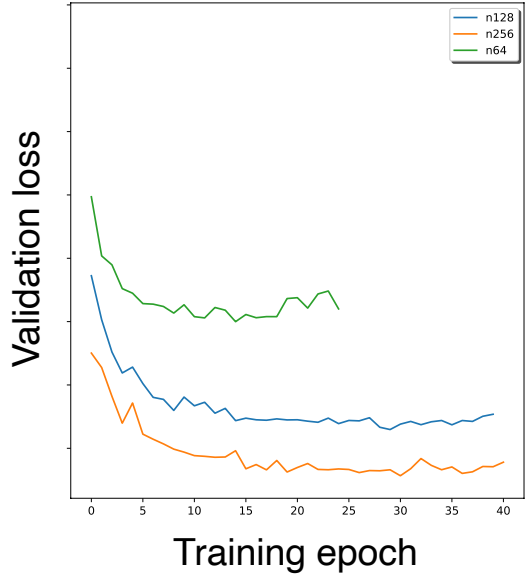

Supplement: S4 Fig — All tests were calculated on simulated examples of the simple bidirectional scenario described in the Methods. (PDF) [file pgen.1010657.s004.pdf]

A) ROC Curve

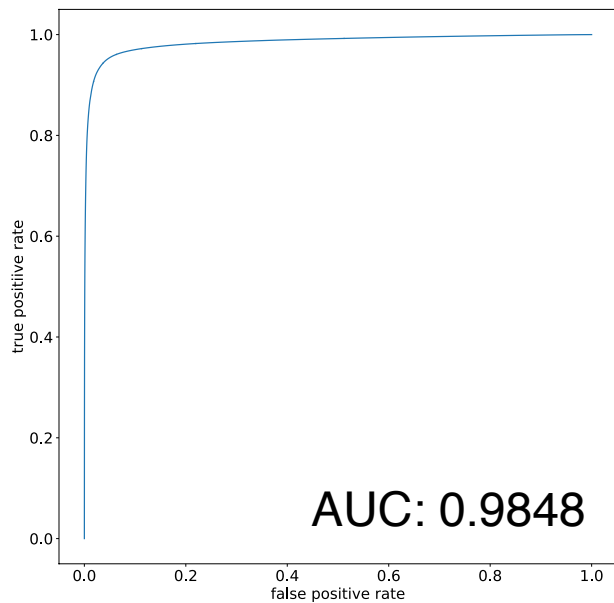

B) Precision-recall curve

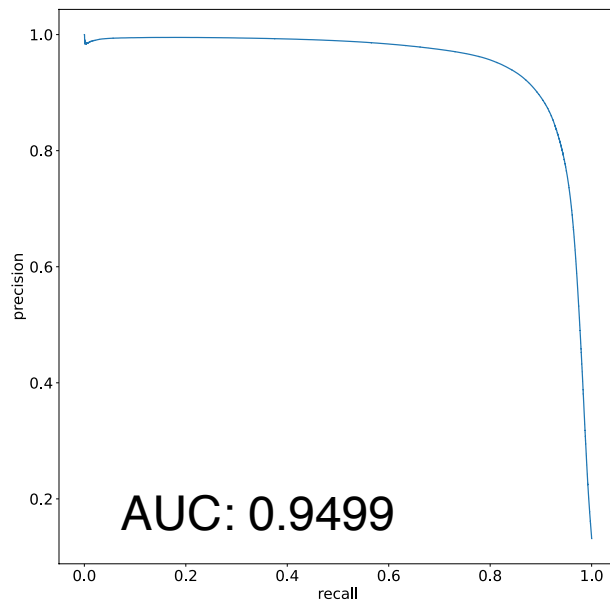

C) Confusion matrix

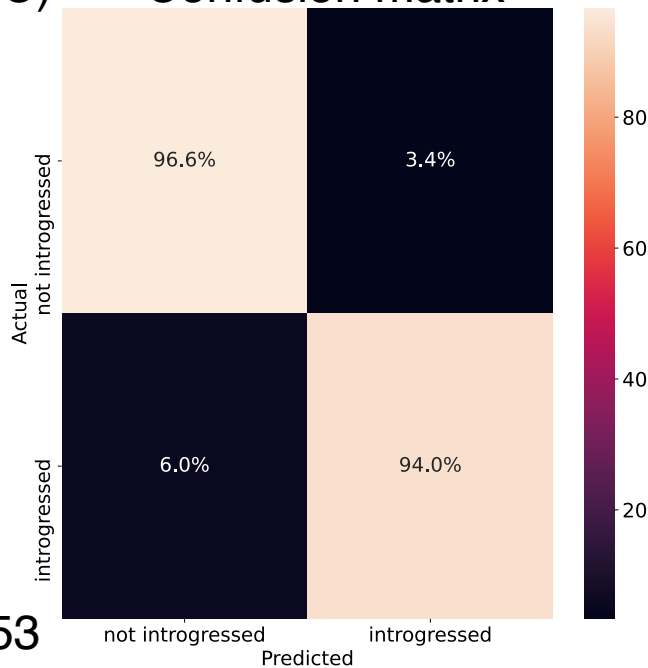

Accuracy: 0.953

Supplement: S6 Fig — The test data here were the same as those examined in Fig 4A, but the network used to perform inference was the same as that used for Fig 4C. (PDF) [file pgen.1010657.s006.pdf]

Deleterious:  $s = -1\text{e-}6$ 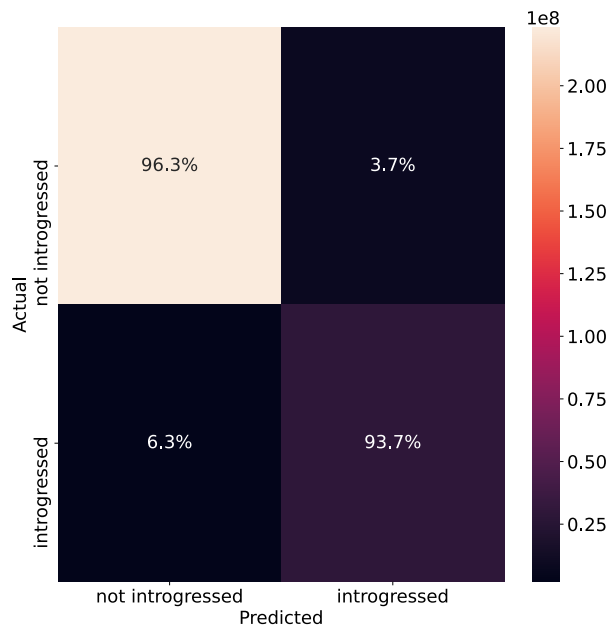Beneficial:  $s = 1\text{e-}6$ 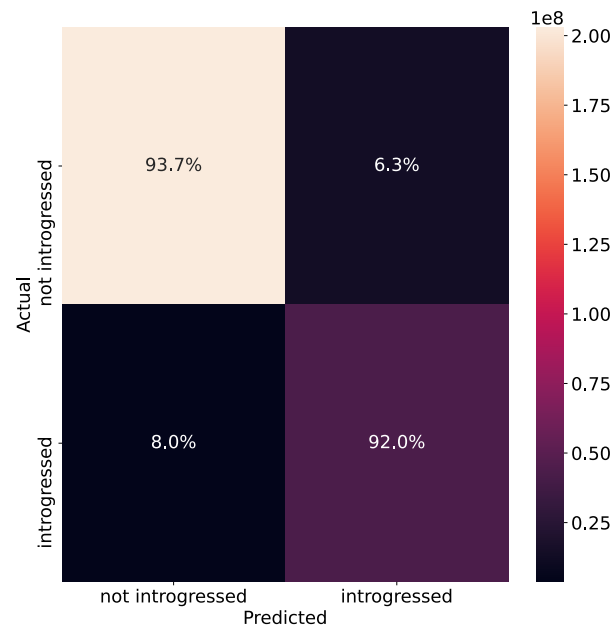Deleterious:  $s = -1\text{e-}5$ 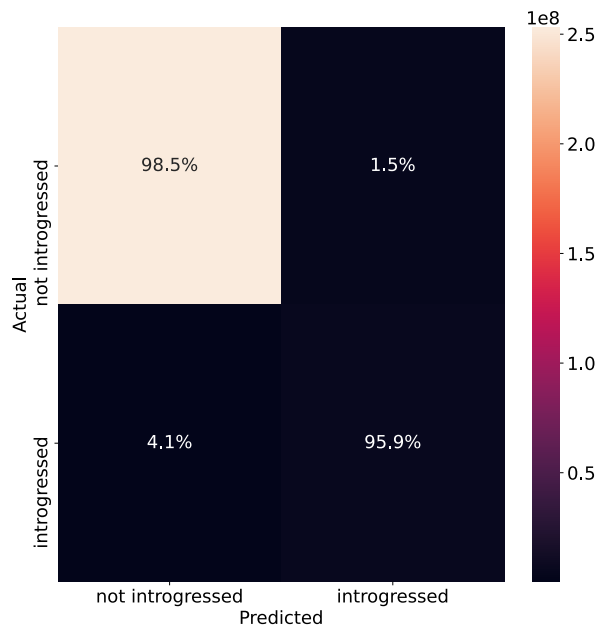Beneficial:  $s = 1\text{e-}5$ 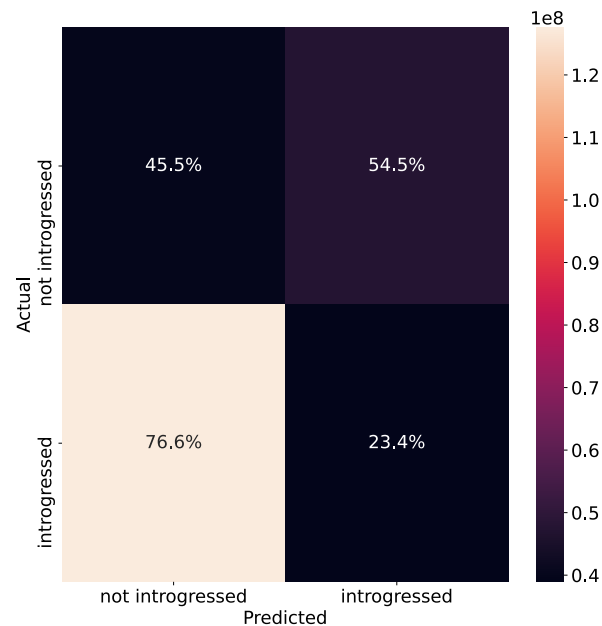

Supplement: S7 Fig — The left column shows confusion matrices obtained when using the same trained network used for the simple bidirectional introgression scenario whose parameters are laid out in Table 1, and applied to data simulated under the same model but with introgressed nucleotides experiencing negative selection. The column on the right shows results when testing the same IntroUNET model on data where introgressed segments are positively selected. The values of s represent the selection coefficient per introgressed nucleotide. Note that the shading represents the number of examples in each entry of the confusion matrix rather than the fraction of examples. (PDF) [file pgen.1010657.s007.pdf]

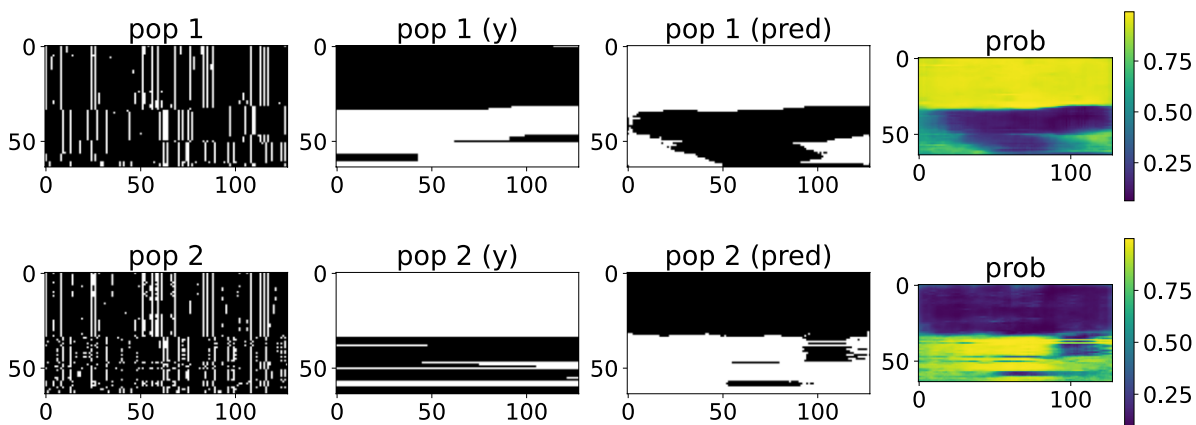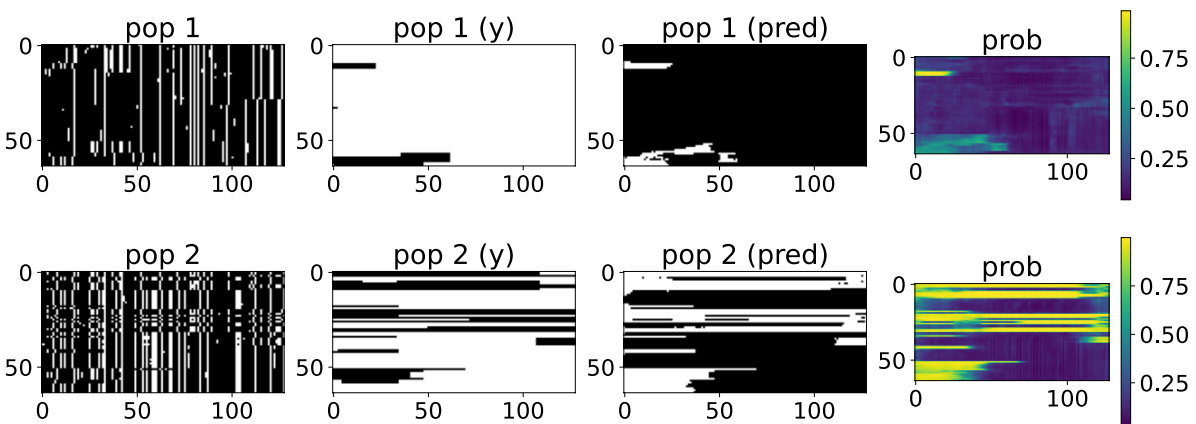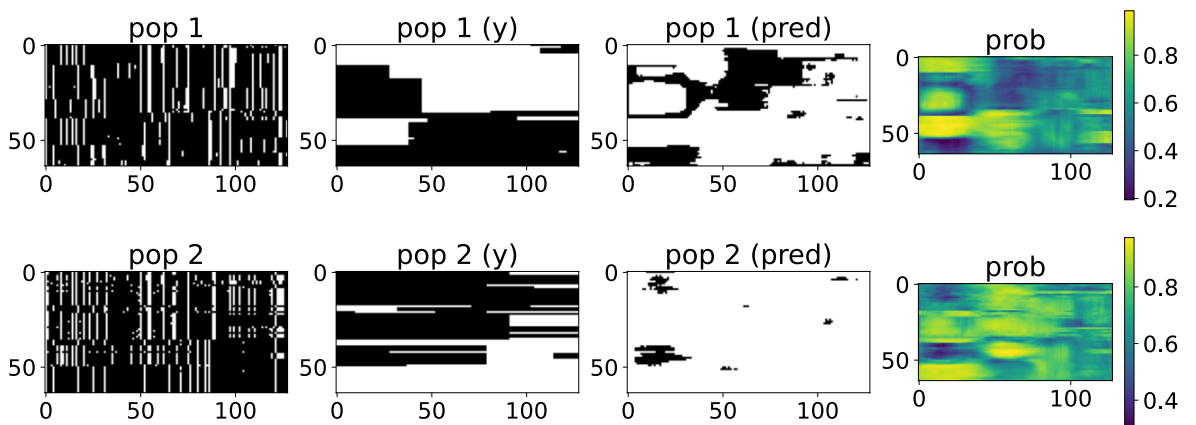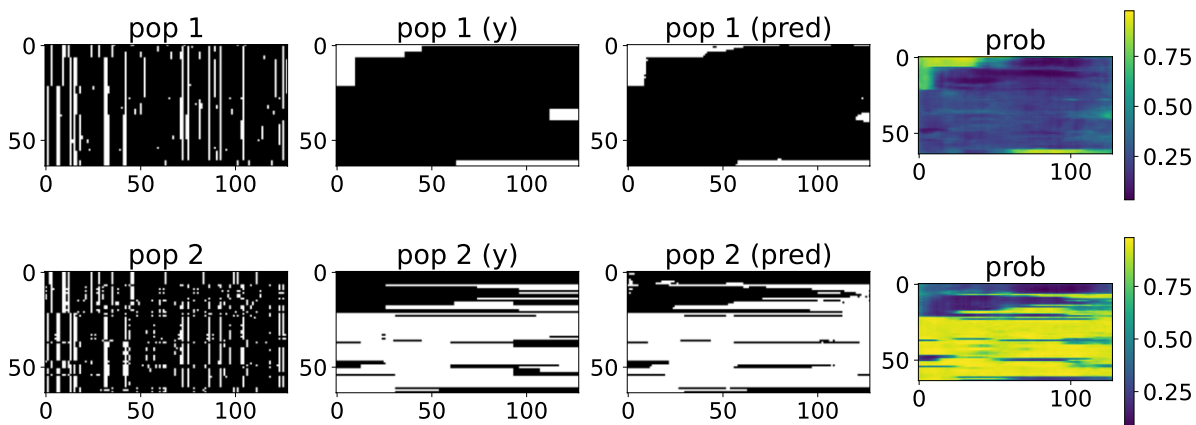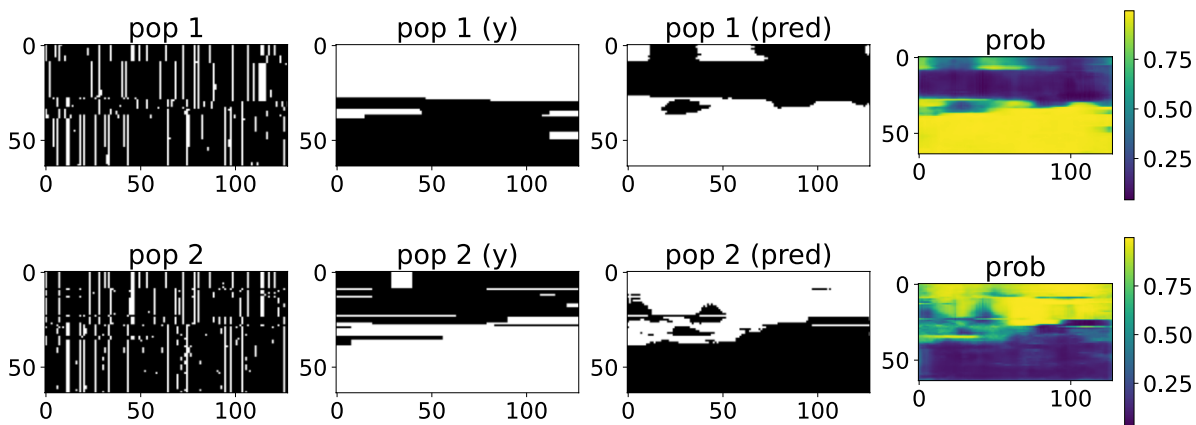

Supplement: S8 Fig — Each example shows the input alignments for the two populations (labeled “pop 1” and “pop 2” respectively), the true introgressed alleles for these two populations (labeled “pop 1 (y)” and “pop 2 (y)” respectively), the introgressed alleles inferred by IntroUNET (“pop 1 (pred)” and “pop 2 (pred)”), and IntroUNET’s inferred introgression probabilities (labelled “prob”). Alignments and introgressed histories, true and predicted, are shown in the same format as in Fig 1. (PDF) [file pgen.1010657.s008.pdf]

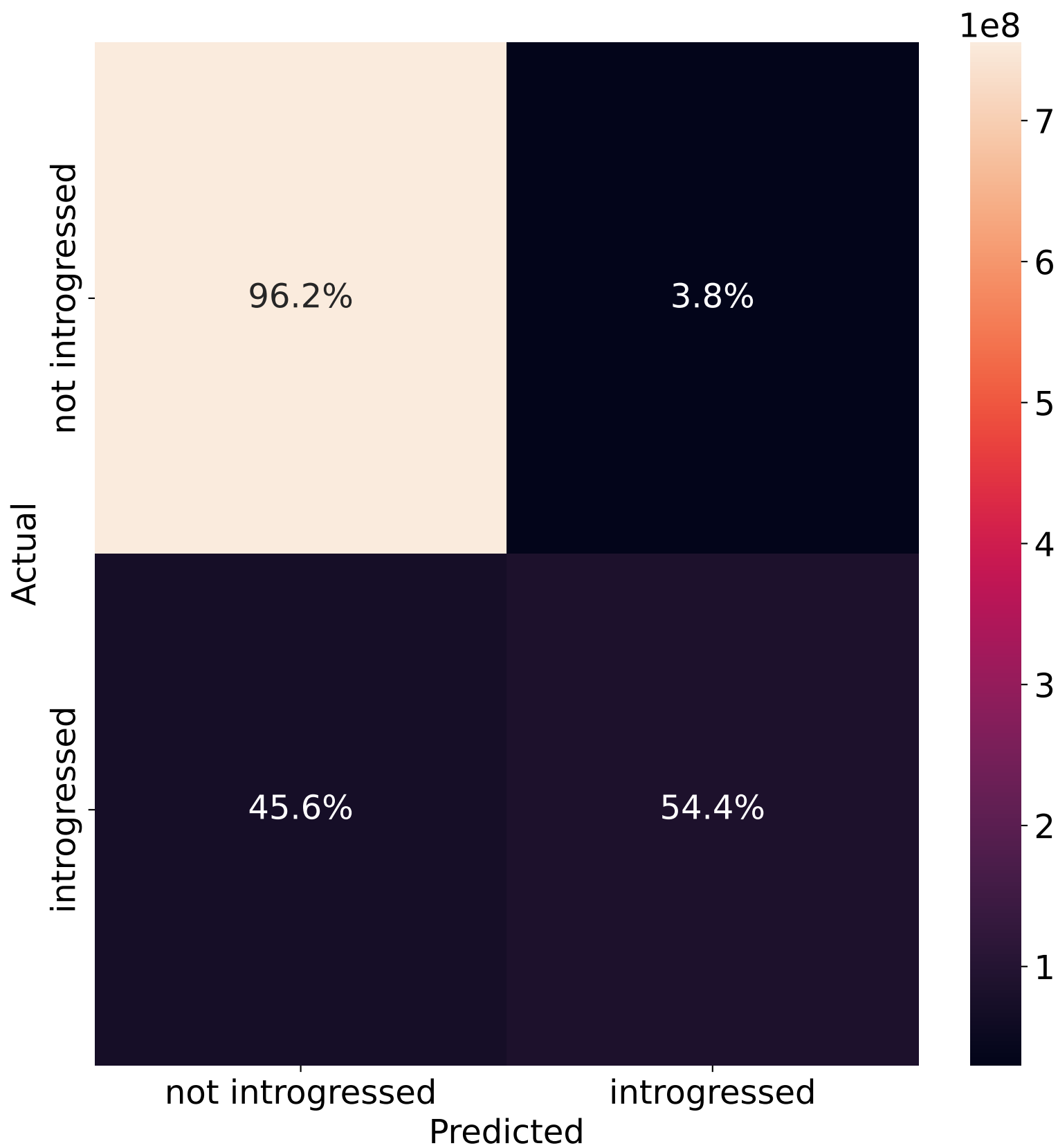

Supplement: S9 Fig — The confusion matrix shows IntroUNET’s classification performance when applying the same trained network used for the simple bidirectional introgression scenario whose parameters are laid out in Table 1 to data simulated under the the D. melanogaster BGS model specified in the Methods. Note that the shading represents the number of examples in each entry of the confusion matrix rather than the fraction of examples. (PDF) [file pgen.1010657.s009.pdf]

Mean  $r$  in central 100 kb

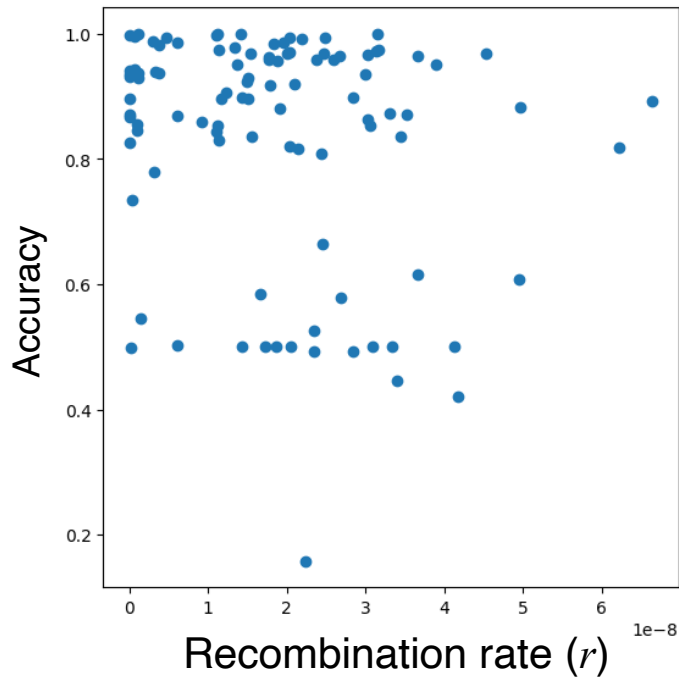

Mean  $r$  in 1 Mb chromosome

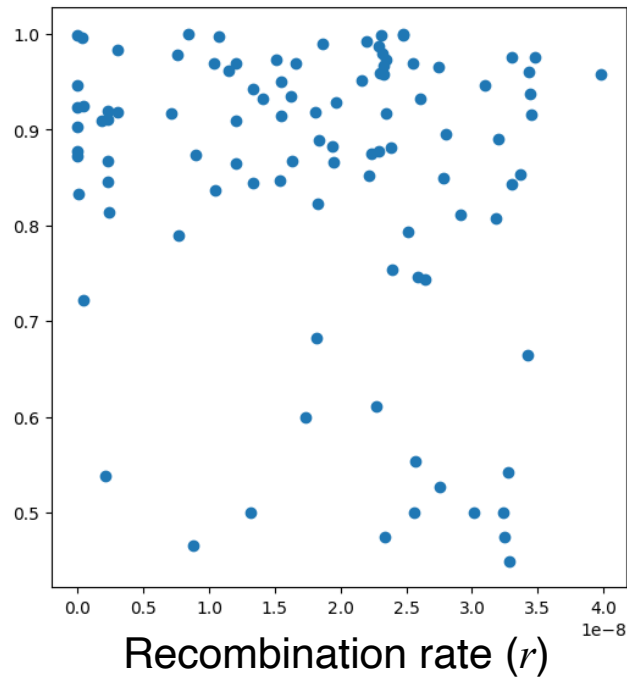

Supplement: S10 Fig — The left panel shows each simulation’s accuracy, averaged across classified pixels from the central 100 kb of the simulated chromosome, as a function of the average recombination rate in that same window. The right panel shows each simulation’s accuracy, averaged across classified pixels from the central 100 kb of the simulated chromosome, as a function of the recombination rate averaged across the entire simulated 1 Mb chromosome. (PDF) [file pgen.1010657.s010.pdf]

A) ROC Curve

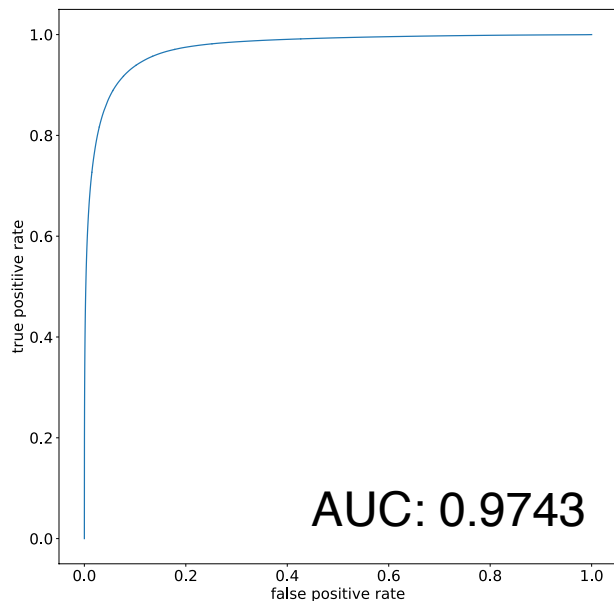

B) Precision-recall curve

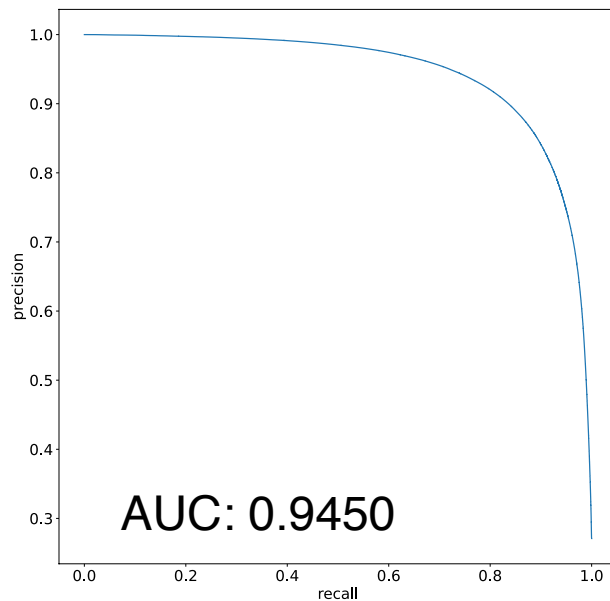

C) Confusion matrix

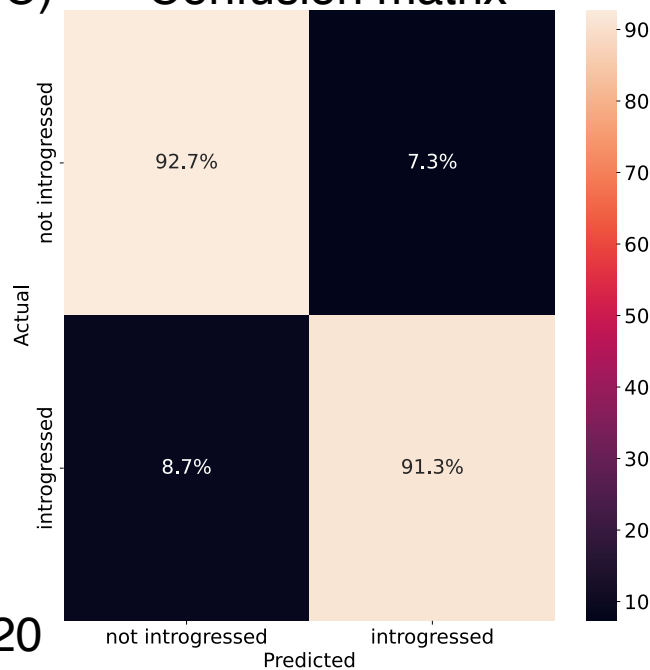

Accuracy: 0.920

Supplement: S11 Fig — The test data here were the same as those examined in Fig 4(C), but here rather than predicted which haplotypes are introgressed, IntroUNET, when given a matrix of diploid genotypes, infers which diploid individuals have at least one introgressed allele at a given polymorphic site. (PDF) [file pgen.1010657.s011.pdf]

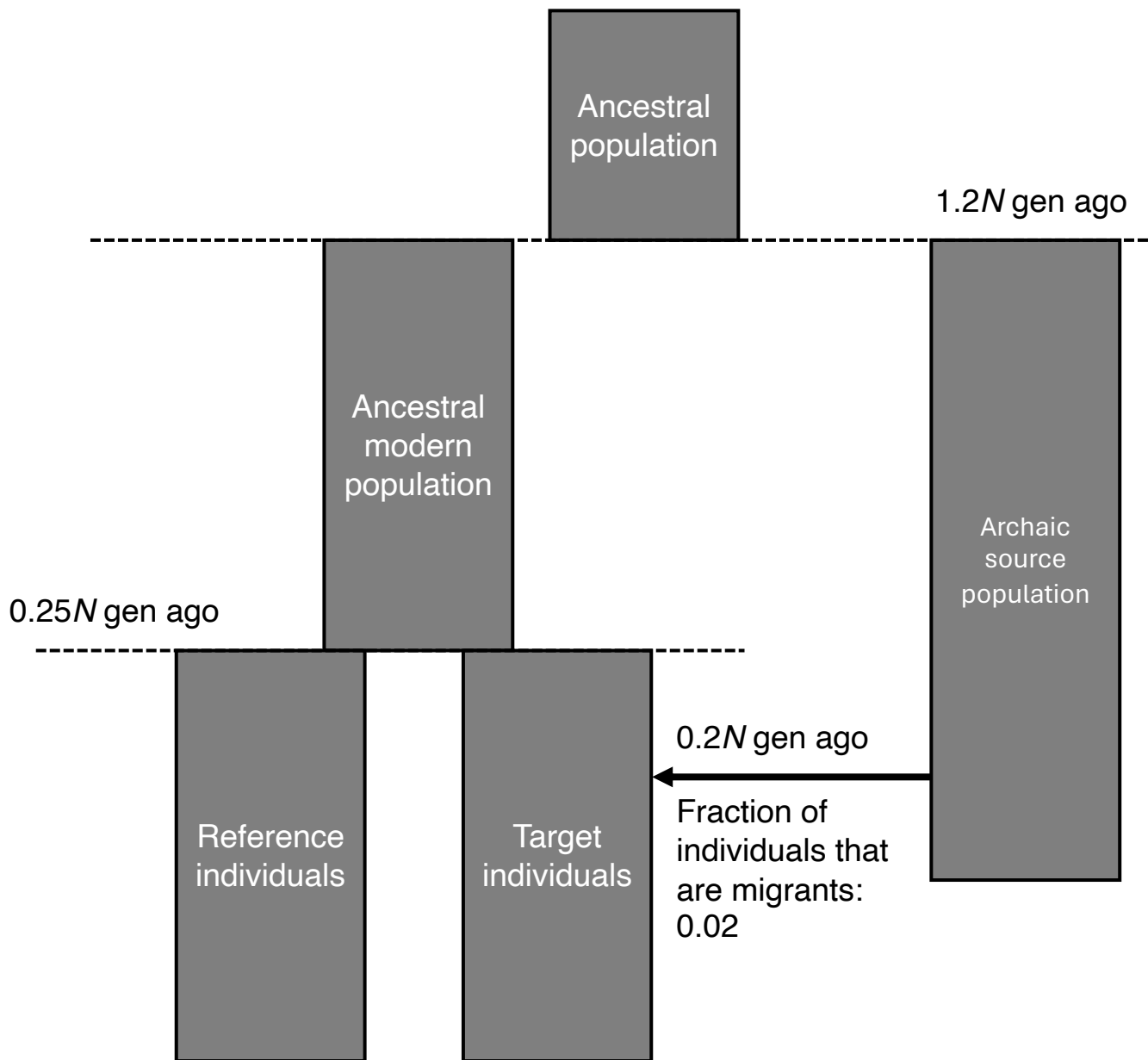

Supplement: S12 Fig — (PDF) [file pgen.1010657.s012.pdf]

# IntroUNET

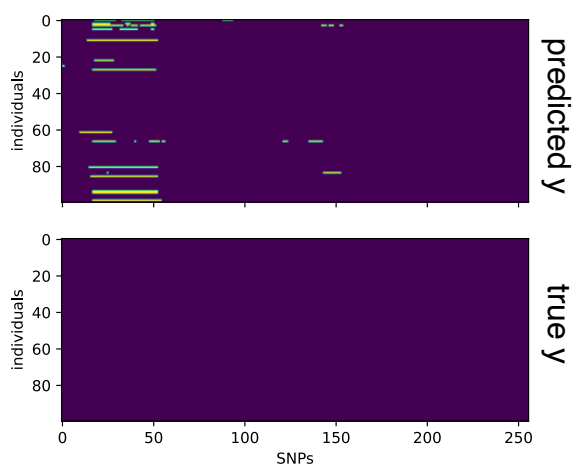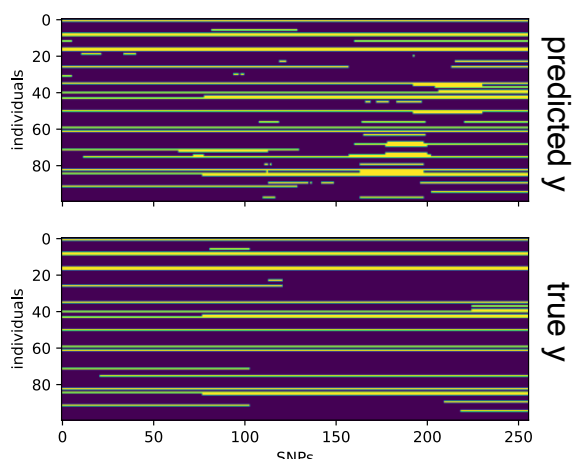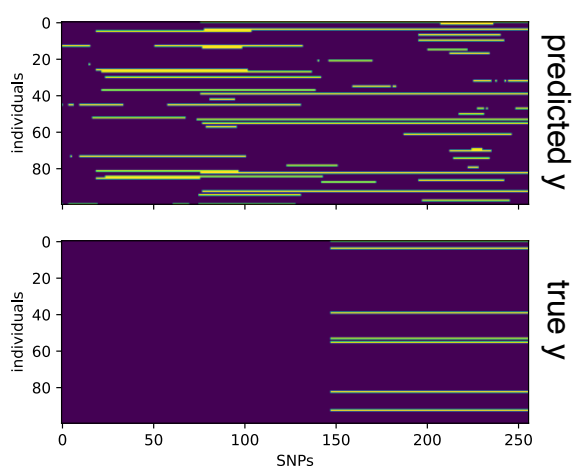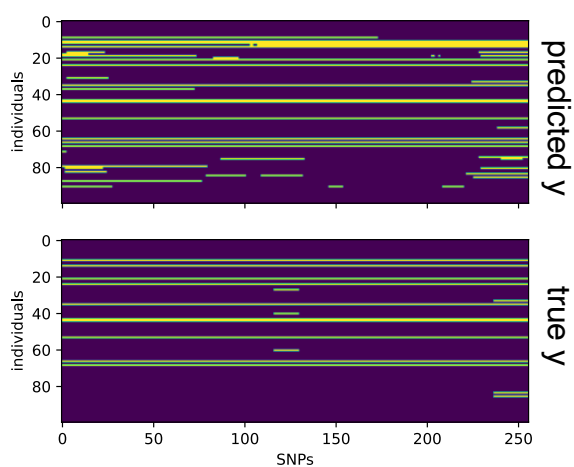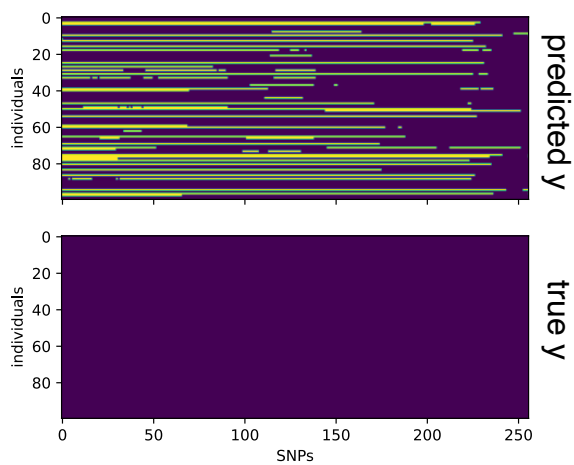

# ArchIE

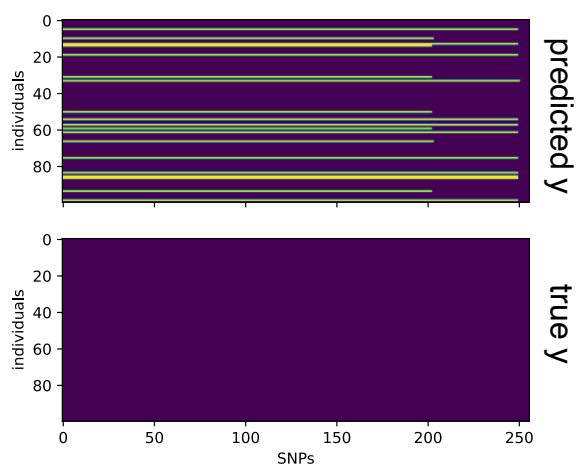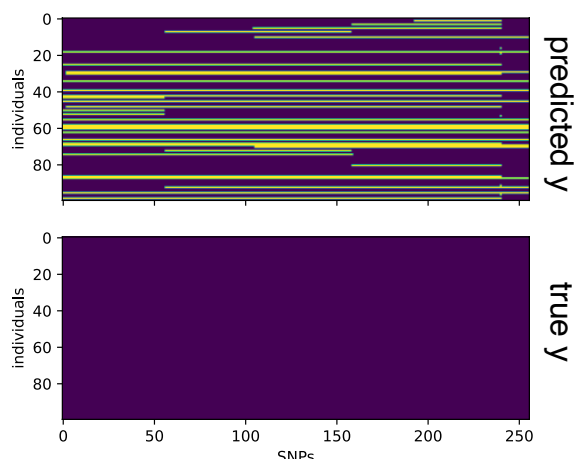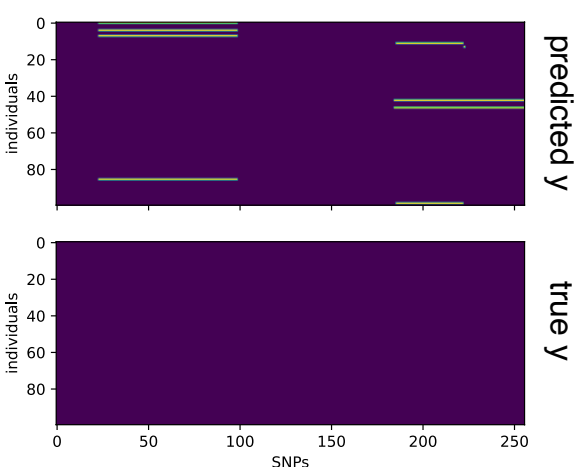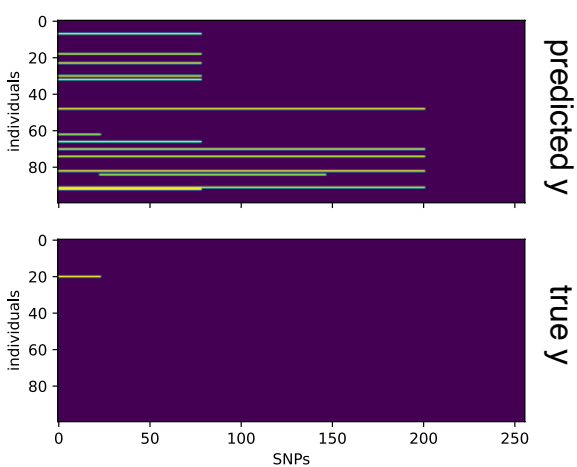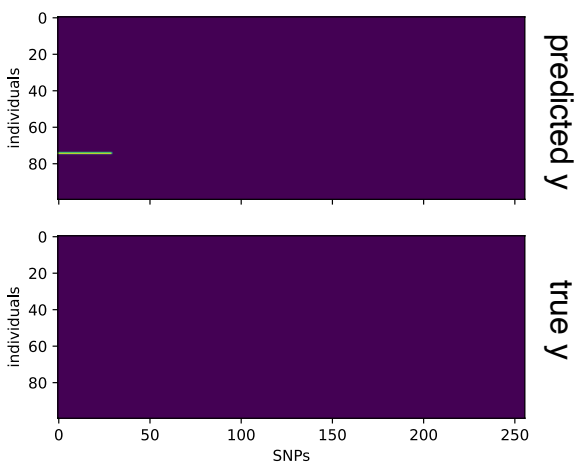

Supplement: S13 Fig — For each example we show the true and inferred introgressed alleles in the recipient population. For each method, both examples with and without introgression are shown. (PDF) [file pgen.1010657.s013.pdf]

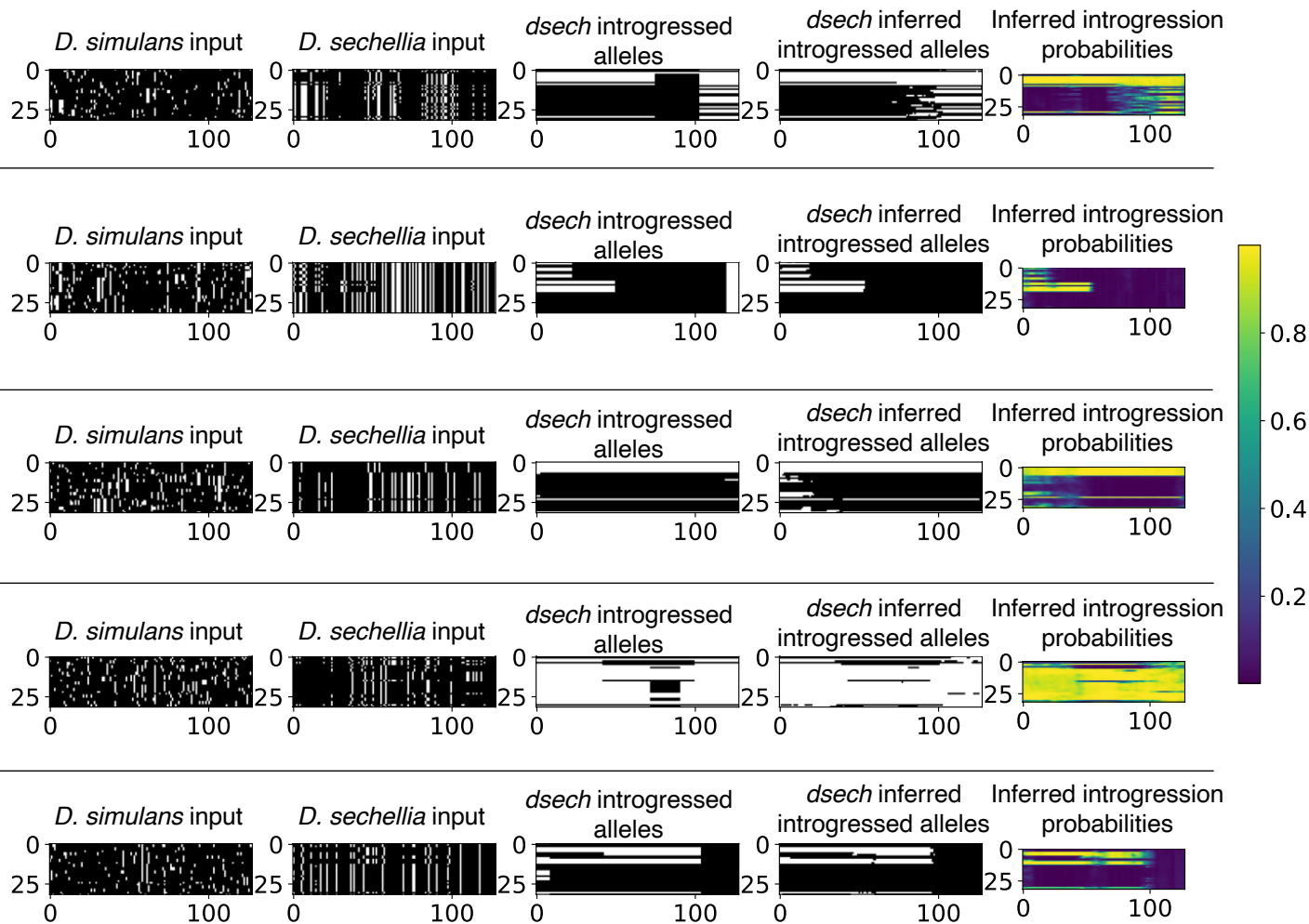

Supplement: S14 Fig — Each example shows the input alignments for the two populations, the true and inferred introgressed alleles for the D. sechellia population, and IntroUNET’s inferred introgression probabilities. Alignments and introgressed histories, true and predicted, are shown in the same format as in Fig 1. (PDF) [file pgen.1010657.s014.pdf]

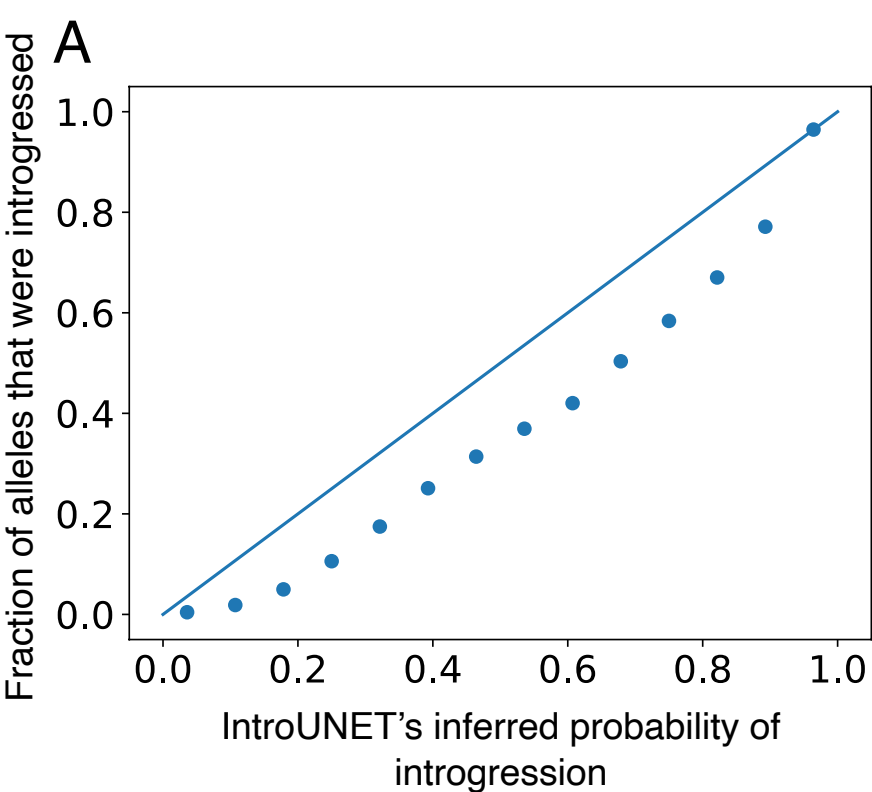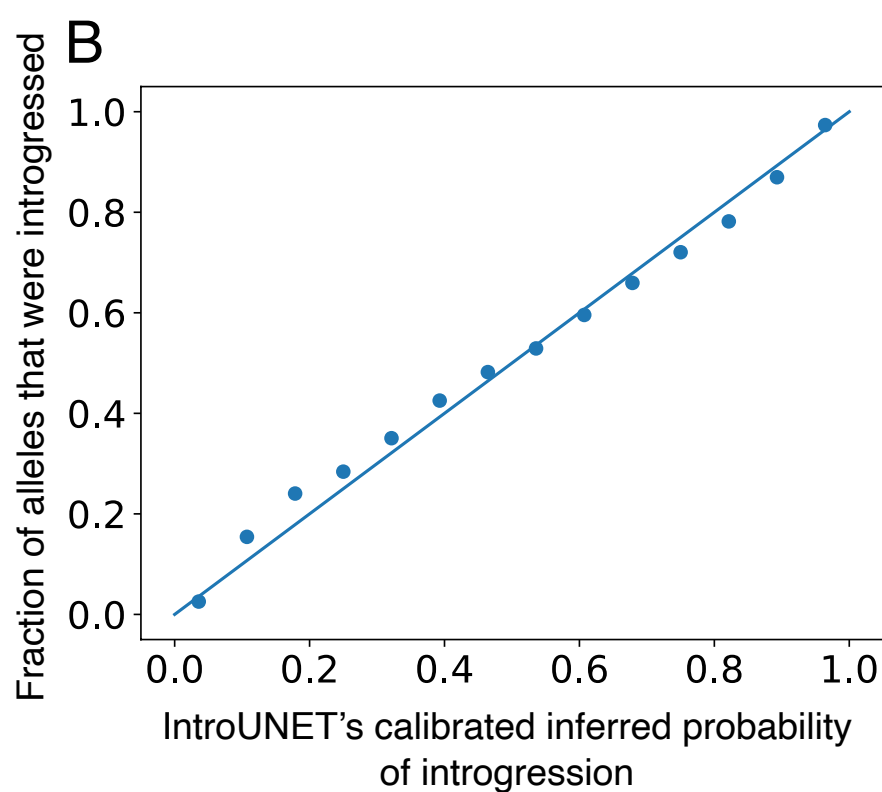

Supplement: S15 Fig — A) The fraction of alleles falling within a given bin of IntroUNET’s predicted probability of introgression that were in fact truly introgressed, prior to Platt recalibration. B) Same as (A), after recalibration. (PDF) [file pgen.1010657.s015.pdf]

## Outside of AI region

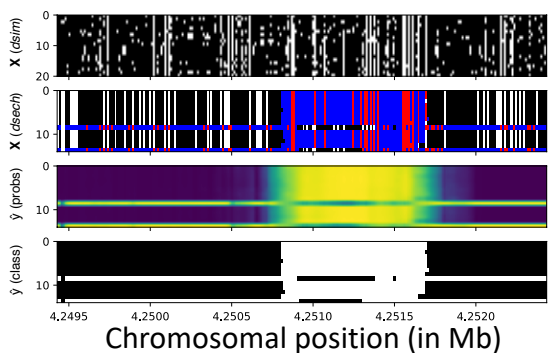

## Within AI region

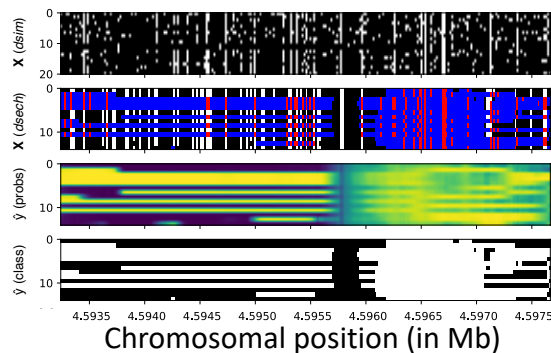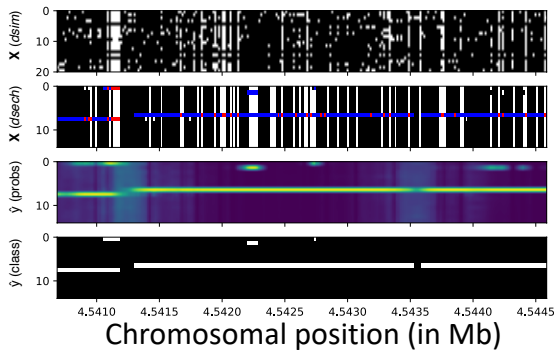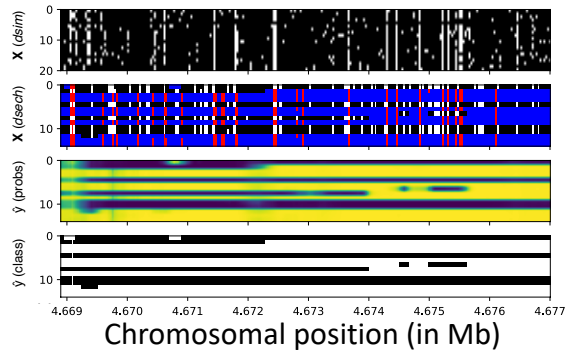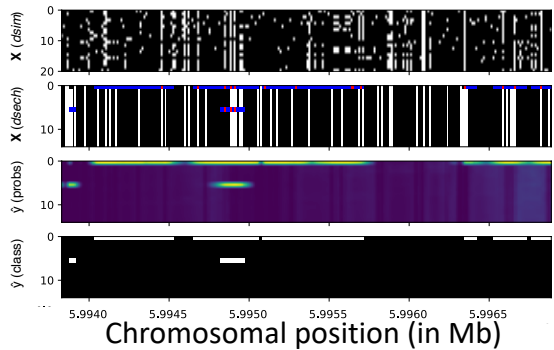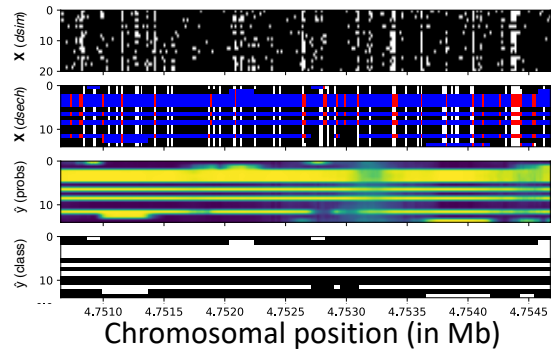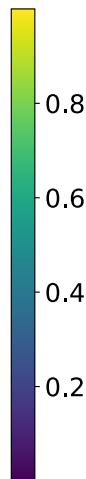

Supplement: S16 Fig — Three of the windows (left) are from outside of the region affected by AI, and the other three (right) are within the AI locus. Each example shows the input alignments for the two populations (labeled “X (dsim)” and “X (dsech)”, respectively), IntroUNET’s inferred introgression probabilities (“y^ (probs)”), and the most probable class for each allele in each individual (“y^ (class)”). Inferred introgressed histories and introgression probabilities are shown in the same format as in Fig 1. Alignments are shown in the same format as for previous figures, with the exception that for D. sechellia which has a different color scheme in haplotypes that were inferred to be introgressed in order to highlight these regions of the alignment: blue for the ancestral allele, and red for the derived allele. (PDF) [file pgen.1010657.s016.pdf]

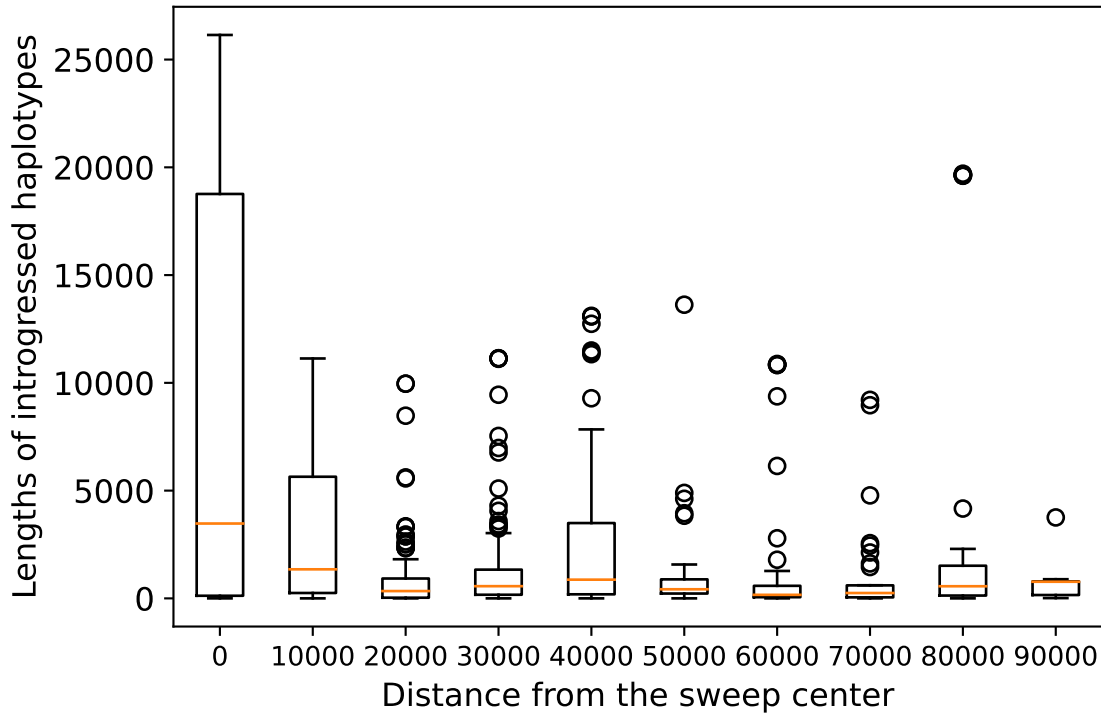

Supplement: S17 Fig — Introgressed haplotypes were defined as runs of consecutive SNPs classified as introgressed for a given individual. The sweep center was set to position 4624900, the center of the window with the lowest level of diversity in this region (data from [39]). (PDF) [file pgen.1010657.s017.pdf]

**A**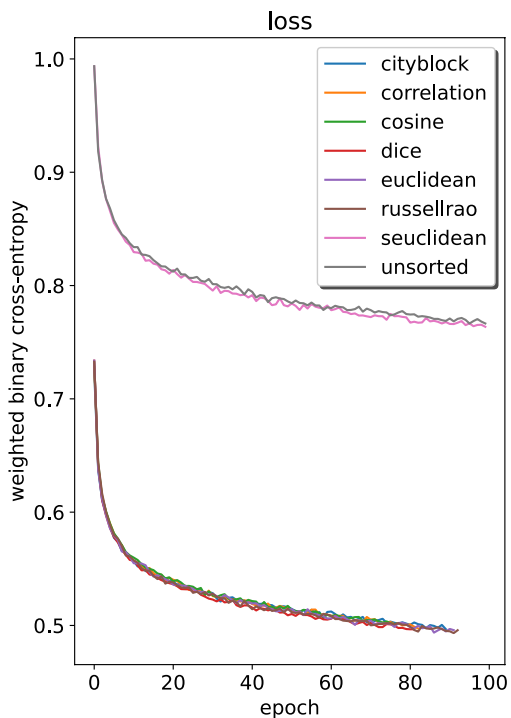**B**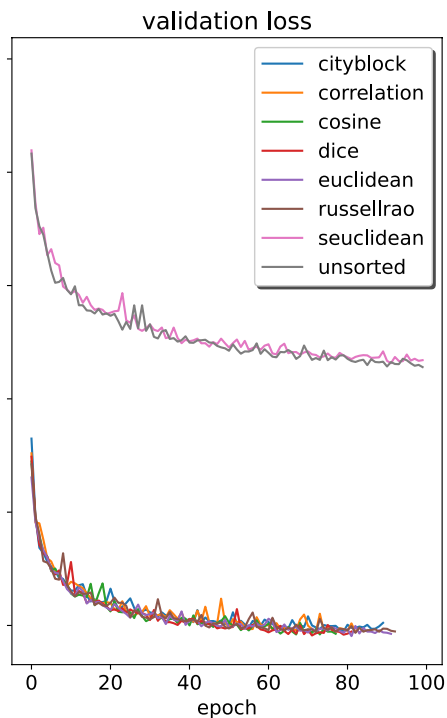**C**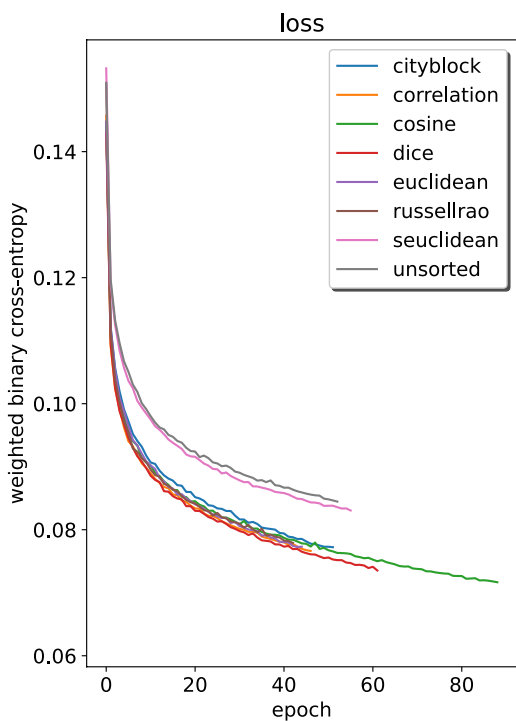**D**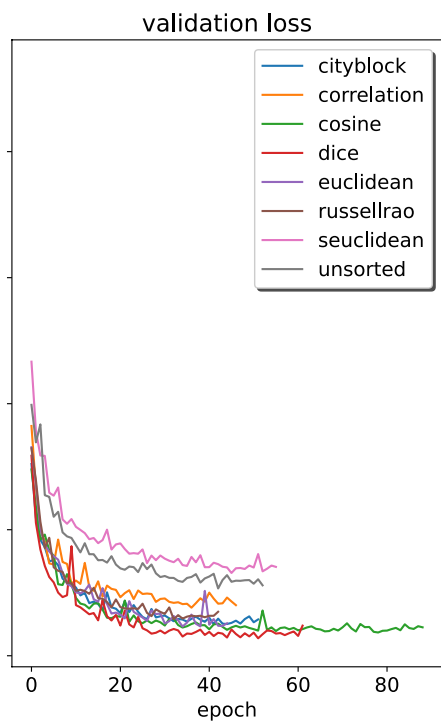

Supplement: S18 Fig — These plots show the values of training (A and C) and validation (B and D) loss over the course of training. Validation loss is usually lower than training in the case of Drosophila because label smoothing was applied to the training data for the purposes of regularization, but not to the validation data. (PDF) [file pgen.1010657.s018.pdf]

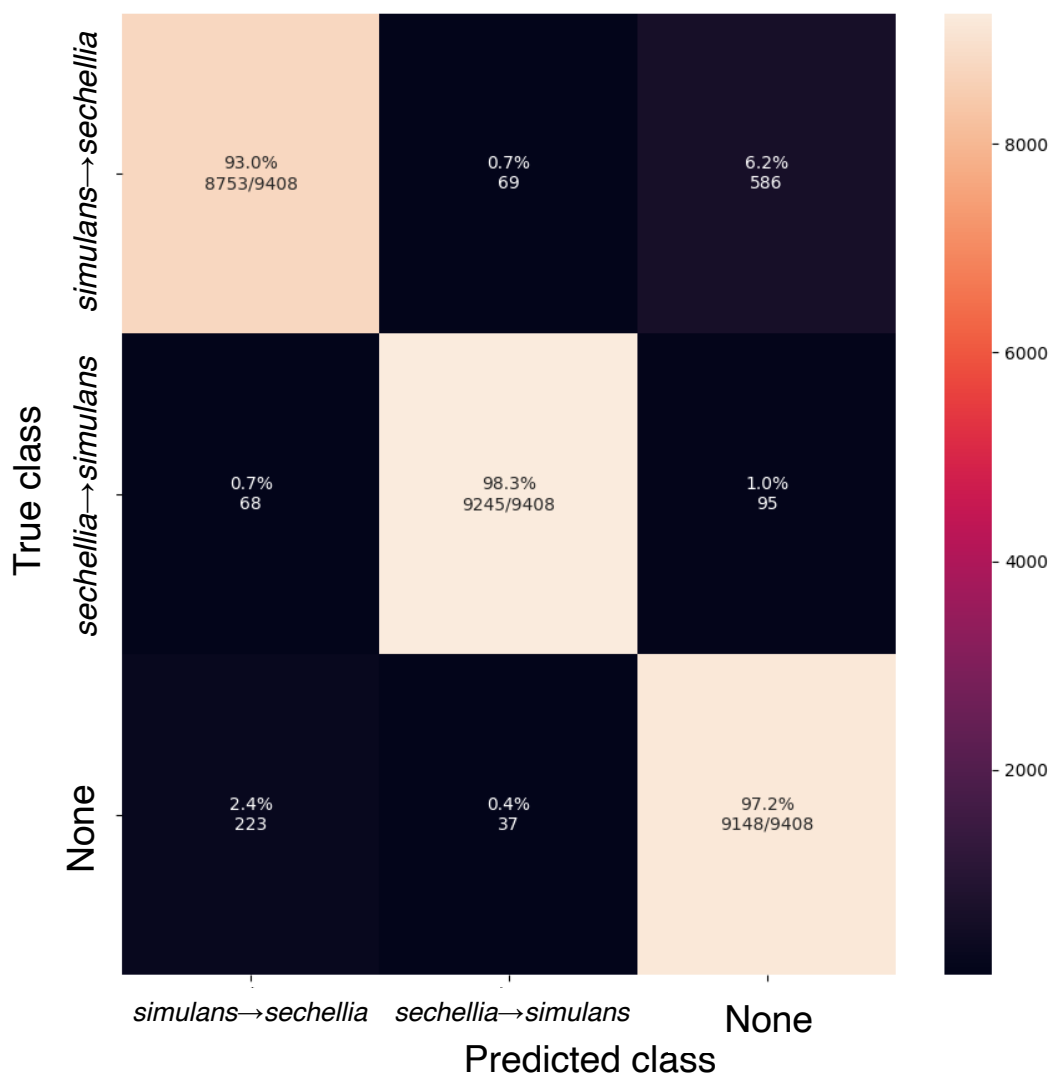

Supplement: S19 Fig — (PDF) [file pgen.1010657.s019.pdf]
