# Supplementary material for: IntroUNET: Identifying introgressed alleles via semantic segmentation
Source: PLoS Genet. 2024 Feb 20;20(2):e1010657. doi: 10.1371/journal.pgen.1010657 (PMC10906877; doi:10.1371/journal.pgen.1010657)
Supplement: S5 Fig — IntroUNET was trained on 25 different combinations of the population split time and the upper bound of the range of possible introgression times (with the lower bound always set to zero). These simulations were performed in the same manner as described for the simple bidirectional model in the Methods, with the exception of these two parameters. Each heatmap in this grid shows the accuracy of one version of IntroUNET on each of the 25 test sets, and the parameter combination used to train that network is marked by a circle. For example, if the true split time is 2N generations ago and the true split time is 0.1 times the split time, one can observe the impact of misspecification on accuracy by comparing the top-left value in the top-left heatmap (i.e. no misspecification in this case) to the top-left value of all other heatmaps in the figure, which experience varying degrees of misspecification. (PDF) [file pgen.1010657.s005.pdf]

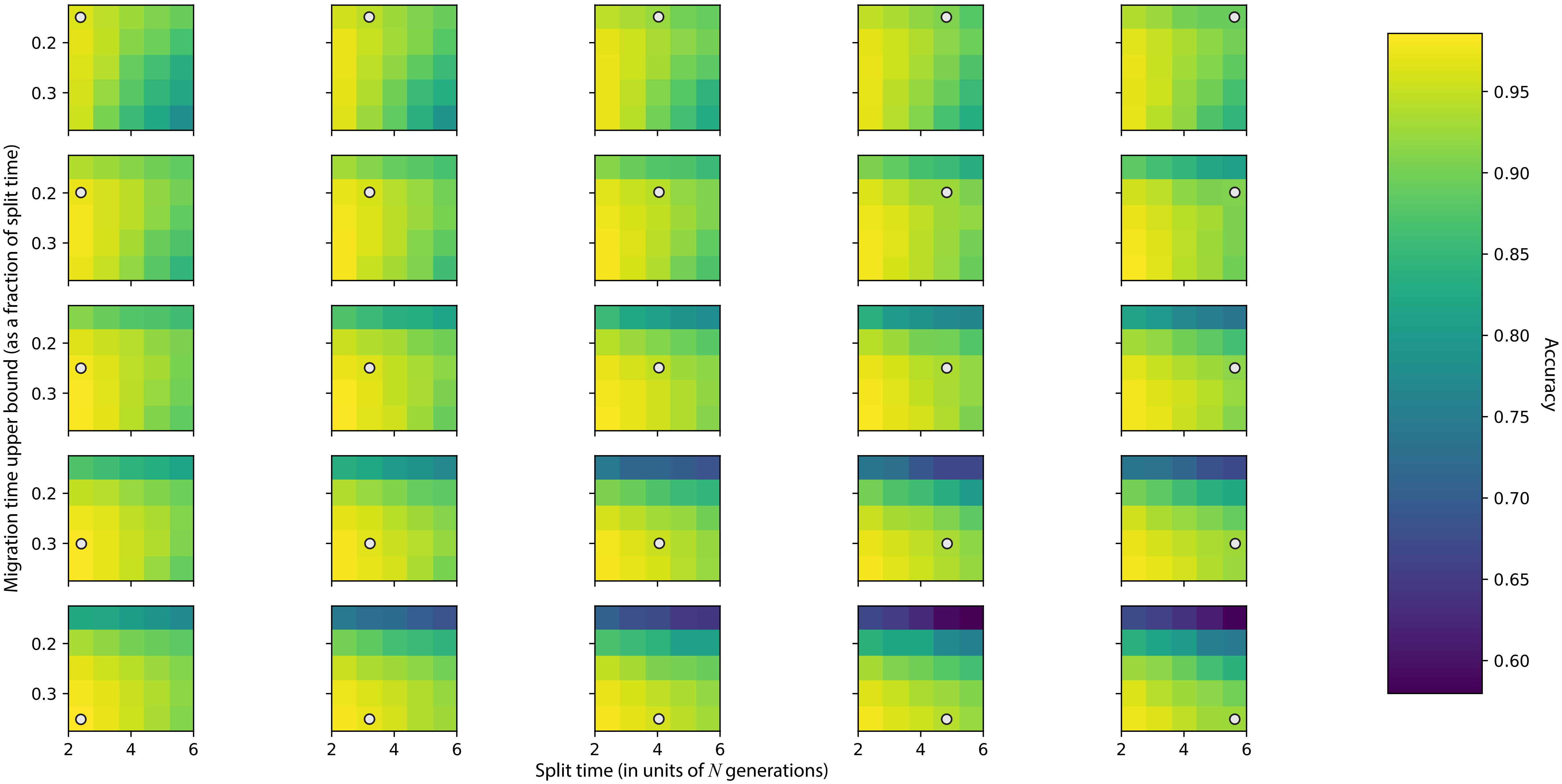

Migration time upper bound (as a fraction of split time)
0.2
0.3
Split time (in units of N generations)
2
4
6
Accuracy
0.95
0.90
0.85
0.80
0.75
0.70
0.65
0.60

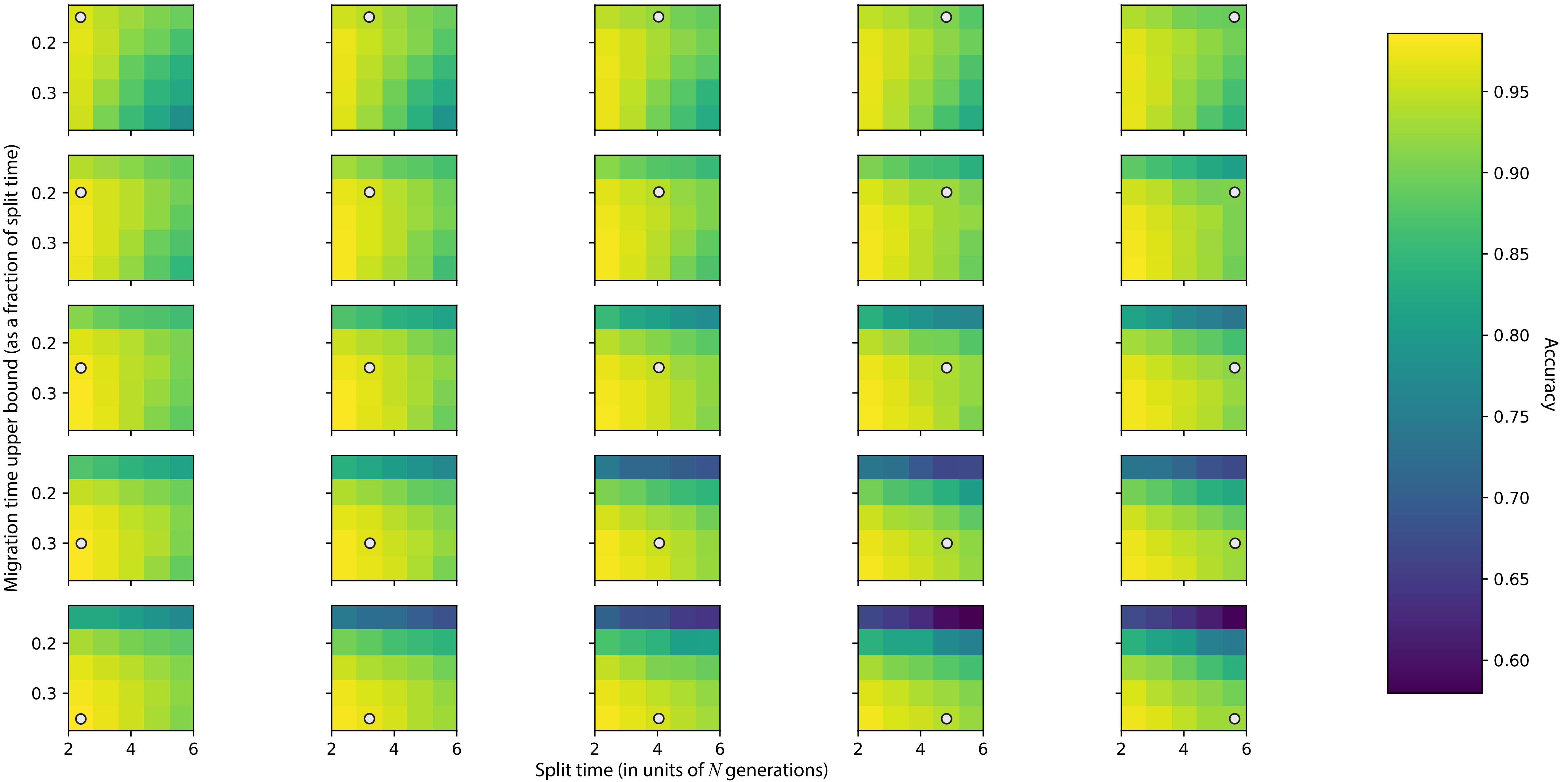
Migration time upper bound (as a fraction of split time)
0.2
0.3
Split time (in units of N generations)
2
4
6
Accuracy
0.95
0.90
0.85
0.80
0.75
0.70
0.65
0.60
