# Supplementary material for: IntroUNET: Identifying introgressed alleles via semantic segmentation
Source: PLoS Genet. 2024 Feb 20;20(2):e1010657. doi: 10.1371/journal.pgen.1010657 (PMC10906877; doi:10.1371/journal.pgen.1010657)
Supplement: S1 Table — The parameters of the model are the ancestral population size (Nref), the final population sizes of D. sechellia and D. simulans (N0−sech and N0−sim), the initial population sizes (Nsech and Nsim), the population split time (ts), and the backwards migration rates (msim → sech and msech → sim). Note that parameter estimates are shown for each bootstrap replicate for which our optimization procedure succeeded (Methods), but only those with log-likelihood scores greater than −1750 were used to simulate training data. (PDF) [file pgen.1010657.s020.pdf]

Log-likelihood	AIC	N_{ref}	N_{0-sech}	N_{0-sim}	N_{sech}	N_{sim}	t_s	$m_{sim\rightarrow sech}$	$m_{sech\rightarrow sim}$
-1.54e+03	3.1e+03	3.27e+05	7.47e+04	3.27e+06	5.94e+06	1.55e+04	5.31e+04	0.0104	0.141
-1.42e+03	2.85e+03	2.86e+05	7.29e+04	2.86e+06	5.55e+06	1.72e+04	5.11e+04	0.0136	0.061
-1.27e+03	2.56e+03	2.94e+05	7.82e+04	2.94e+06	5.79e+06	1.75e+04	5.4e+04	0.0138	0.05
-1.28e+03	2.58e+03	3.04e+05	7.81e+04	3.04e+06	5.46e+06	1.79e+04	5.36e+04	0.0143	0.0648
-1.26e+03	2.54e+03	2.88e+05	7.42e+04	2.88e+06	5.14e+06	1.73e+04	5.12e+04	0.0133	0.0595
-1.27e+03	2.55e+03	3.12e+05	8.1e+04	3.12e+06	5.7e+06	1.8e+04	5.53e+04	0.0141	0.0694
-2.99e+03	5.99e+03	3.56e+04	2.54e+04	2.02e+05	3.43e+06	1.84e+04	9.49e+04	0.00216	0.0204
-1.15e+03	2.32e+03	2.91e+05	7.67e+04	2.91e+06	5.16e+06	1.65e+04	5.24e+04	0.0129	0.0528
-1.07e+03	2.15e+03	2.84e+05	7.16e+04	2.84e+06	5.16e+06	1.62e+04	5.06e+04	0.0132	0.0589
-1.64e+03	3.29e+03	2.91e+05	7.25e+04	2.93e+05	5.15e+06	2.06e+04	5.51e+04	0.0116	0.103
-1.4e+03	2.8e+03	3.04e+05	7.6e+04	3.04e+06	5.32e+06	1.72e+04	5.31e+04	0.0136	0.0734
-2.98e+03	5.97e+03	3.64e+04	2.06e+04	2.25e+05	3.48e+06	1.77e+04	9.71e+04	0.00216	0.0239
-1.2e+03	2.41e+03	2.91e+05	7.23e+04	2.9e+06	5.05e+06	1.66e+04	5.09e+04	0.0132	0.078
-1.11e+03	2.23e+03	3.04e+05	8.15e+04	3.04e+06	5.67e+06	1.73e+04	5.62e+04	0.0137	0.0675
-1.25e+03	2.51e+03	2.94e+05	7.71e+04	2.94e+06	5.5e+06	1.72e+04	5.38e+04	0.013	0.0571
-1.31e+03	2.64e+03	2.95e+05	7.98e+04	2.95e+06	5.42e+06	1.69e+04	5.34e+04	0.0134	0.0529
-1.14e+03	2.29e+03	2.73e+05	6.64e+04	2.73e+06	5.14e+06	1.56e+04	4.78e+04	0.0131	0.0573
-1.23e+03	2.48e+03	2.95e+05	7.16e+04	2.95e+06	5.44e+06	1.72e+04	5.06e+04	0.0137	0.0626
-3.13e+03	6.27e+03	3.62e+04	2.42e+04	2.84e+05	3.46e+06	1.79e+04	9.44e+04	0.00223	0.0225
-2.96e+03	5.94e+03	4.24e+04	2.53e+04	2.99e+05	3.58e+06	1.84e+04	1.01e+05	0.00247	0.0255
-2.89e+03	5.79e+03	3.95e+04	2.42e+04	6.15e+04	3.6e+06	1.79e+04	9.48e+04	0.0024	0.0213
-1.24e+03	2.49e+03	2.94e+05	7.79e+04	2.94e+06	5.82e+06	1.66e+04	5.38e+04	0.013	0.0662
-1.43e+03	2.88e+03	2.98e+05	7.5e+04	2.98e+06	5.47e+06	1.77e+04	5.17e+04	0.0144	0.0688
-1.29e+03	2.6e+03	3.08e+05	7.78e+04	3.08e+06	5.69e+06	1.74e+04	5.55e+04	0.0132	0.0848
-1.33e+03	2.66e+03	3.01e+05	7.75e+04	3.01e+06	5.66e+06	1.74e+04	5.3e+04	0.0137	0.0567
-1.27e+03	2.56e+03	2.87e+05	7.73e+04	2.87e+06	5.05e+06	1.66e+04	5.19e+04	0.0133	0.0649
-2.88e+03	5.78e+03	3.87e+04	2.57e+04	3.87e+05	3.44e+06	1.86e+04	9.52e+04	0.00236	0.0231
-1.31e+03	2.62e+03	3e+05	7.83e+04	3e+06	5.68e+06	1.71e+04	5.43e+04	0.0136	0.0767
-1.26e+03	2.53e+03	3.05e+05	8.19e+04	3.05e+06	5.85e+06	1.78e+04	5.47e+04	0.0135	0.0542
-1.34e+03	2.69e+03	3.01e+05	7.67e+04	3.01e+06	5.65e+06	1.75e+04	5.31e+04	0.0134	0.0666
-1.35e+03	2.72e+03	2.94e+05	7.76e+04	2.94e+06	5.47e+06	1.72e+04	5.29e+04	0.0131	0.0654
-2.68e+03	5.38e+03	3.72e+04	2.5e+04	3.72e+05	3.72e+06	1.91e+04	9.91e+04	0.00239	0.0189
-1.28e+03	2.57e+03	2.81e+05	7.61e+04	2.81e+06	5.43e+06	1.66e+04	5.15e+04	0.0134	0.0642
-2.84e+03	5.69e+03	3.66e+04	2.58e+04	3.11e+05	3.52e+06	1.79e+04	9.72e+04	0.00221	0.0195
-1.18e+03	2.36e+03	2.93e+05	7.66e+04	2.93e+06	5.32e+06	1.65e+04	5.23e+04	0.0134	0.0628
-1.26e+03	2.54e+03	2.9e+05	7.13e+04	2.9e+06	5.42e+06	1.65e+04	5.01e+04	0.0133	0.0538
-3e+03	6.01e+03	4.94e+04	2.75e+04	4.94e+05	3.29e+06	1.74e+04	9.03e+04	0.00318	0.028
-1.24e+03	2.49e+03	2.92e+05	7.67e+04	2.92e+06	5.66e+06	1.74e+04	5.24e+04	0.0139	0.0415
-1.35e+03	2.71e+03	3.04e+05	7.74e+04	3.04e+06	5.33e+06	1.7e+04	5.32e+04	0.0138	0.0694
-2.51e+03	5.04e+03	3.57e+04	2.37e+04	1.51e+05	3.47e+06	1.66e+04	9.52e+04	0.00231	0.0225
-1.23e+03	2.48e+03	2.8e+05	7.53e+04	2.8e+06	5.55e+06	1.67e+04	5.18e+04	0.0127	0.0531
-1.35e+03	2.72e+03	2.9e+05	7.16e+04	2.9e+06	5.29e+06	1.65e+04	5.18e+04	0.0134	0.082
-2.72e+03	5.45e+03	3.61e+04	2.45e+04	3.39e+05	3.55e+06	1.81e+04	9.61e+04	0.00218	0.0196
-1.22e+03	2.46e+03	3.04e+05	7.81e+04	3.04e+06	5.38e+06	1.77e+04	5.51e+04	0.0139	0.0688
-1.26e+03	2.53e+03	3.04e+05	7.64e+04	3.04e+06	5.86e+06	1.78e+04	5.5e+04	0.013	0.0623
-1.71e+03	3.44e+03	3.27e+05	7.92e+04	3.27e+06	5.2e+06	1.92e+04	5.03e+04	0.0145	0.00148
-1.74e+03	3.5e+03	2.21e+05	9.07e+04	2.21e+06	5.34e+06	1.89e+04	5.43e+04	0.0128	0.0317
-2.68e+03	5.38e+03	3.4e+04	2.81e+04	2e+05	3.2e+06	1.84e+04	9.06e+04	0.00232	0.0191
-2.72e+03	5.45e+03	3.69e+04	2.3e+04	8.31e+04	3.39e+06	1.76e+04	9.06e+04	0.00233	0.022
-1.25e+03	2.51e+03	2.93e+05	7.76e+04	2.93e+06	5.43e+06	1.7e+04	5.19e+04	0.0138	0.0555
-1.1e+03	2.22e+03	3.09e+05	7.59e+04	3.09e+06	5.76e+06	1.72e+04	5.47e+04	0.0133	0.0786
-1.19e+03	2.39e+03	2.94e+05	7.38e+04	2.94e+06	5.64e+06	1.62e+04	5.22e+04	0.0137	0.0796
-2.81e+03	5.64e+03	1.49e+05	4.99e+04	2.64e+04	3.46e+06	1.62e+04	7.95e+04	0.00791	0.0968
-1.28e+03	2.58e+03	2.95e+05	7.57e+04	2.95e+06	5.31e+06	1.69e+04	5.26e+04	0.0137	0.0771
-1.25e+03	2.51e+03	3e+05	7.56e+04	3e+06	5.6e+06	1.7e+04	5.27e+04	0.0135	0.0696
-1.33e+03	2.67e+03	2.98e+05	8.09e+04	2.98e+06	5.84e+06	1.78e+04	5.51e+04	0.0139	0.0645
-1.03e+03	2.08e+03	2.92e+05	7.14e+04	2.56e+06	5.51e+06	1.62e+04	5.19e+04	0.0134	0.0801

Table S1: Bootstrap parameter estimates for the *D. simulans* and *D. sechellia* joint demographic model obtained via ∂a∂i. The parameters of the model are the ancestral population size (N_{ref}), the final population sizes of *D. sechellia* and *D. simulans* (N_{0-sech} and N_{0-sim}), the initial population sizes (N_{sech} and N_{sim}), the population split time (t_s), and the backwards migration rates ($m_{sim\rightarrow sech}$ and $m_{sech\rightarrow sim}$). Note that parameter estimates are shown for each bootstrap replicate for which our optimization procedure succeeded (Methods), but only those with log-likelihood scores greater than -1750 were used to simulate training data.
