# Supplementary material for: IntroUNET: Identifying introgressed alleles via semantic segmentation
Source: PLoS Genet. 2024 Feb 20;20(2):e1010657. doi: 10.1371/journal.pgen.1010657 (PMC10906877; doi:10.1371/journal.pgen.1010657)
Supplement: S2 Table — The simulation and formatting results for this table were computed from a small sample of 430 replicates over 4 cores of an Intel Core i9–9900K CPU @ 3.60GHz and then scaled to give estimates for 105 replicates in each case. The Formatting column lists the time estimates for alignment sorting (for IntroUNET) and statistic calculation (for ArchIE). The Discriminator and Segmenter columns list the training times for classifying entire windows and for identifying introgressed haplotypes, respectively. In the GPU columns the estimate is simply the run time for the training described. The training of the neural networks was done on an NVIDIA A40 GPU, and we found that VRAM usage was <12Gb in all cases. We note that the ArchIE method computes statistics over the entire simulated window (224.64 on average in our simulated 50kb windows) whereas our method only formats a small sequential sample of SNPs from each replicate (192 for the Archaic introgression program). Below the time estimates for simulation are the simulated region size, and below the formatting times are the resulting “image” size or (populations, individuals, sites) and for the case of ArchIE, the window size in base pairs. Note that we do not include the time for execution on data after training, but we observed that classification times for all are generally negligible (although the sorting/statistic calculation steps must be performed first and these can be costly as shown in the Formatting section). (PDF) [file pgen.1010657.s021.pdf]

Method and problem	Simulation (100k sims)	Formatting	Discriminator training	Segmenter training
`IntroUNET`: simple bidirectional scenario	100.1 h<br>110kb	29 h<br>(2, 64, 128)	0.6 h	4.6 h
`IntroUNET`: Drosophila simulans-sechellia model	4.9 h<br>10kb	21.2 h<br>(2, 32, 128)	0.9 h	3.7 h
`IntroUNET`: Archaic introgression model	10.7 h<br>1Mb	48.5 h<br>(2, 112, 192)	N/A	8.3 h
`ArchIE`: Archaic introgression model	10.7 h	107.4 h<br>50kb	N/A	7 min

Table S2: CPU / GPU time estimates for accomplishing the experiments in the paper. The simulation and formatting results for this table were computed from a small sample of 430 replicates over 4 cores of an Intel Core i9-9900K CPU @ 3.60GHz and then scaled to give estimates for 10^5 replicates in each case. The Formatting column lists the time estimates for alignment sorting (for `IntroUNET`) and statistic calculation (for `ArchIE`). The Discriminator and Segmenter columns list the training times for classifying entire windows and for identifying introgressed haplotypes, respectively. In the GPU columns the estimate is simply the run time for the training described. The training of the neural networks was done on an NVIDIA A40 GPU, and we found that VRAM usage was <12Gb in all cases. We note that the `ArchIE` method computes statistics over the entire simulated window (224.64 on average in our simulated 50kb windows) whereas our method only formats a small sequential sample of SNPs from each replicate (192 for the Archaic introgression program). Below the time estimates for simulation are the simulated region size, and below the formatting times are the resulting "image" size or (populations, individuals, sites) and for the case of `ArchIE`, the window size in base pairs. Note that we do not include the time for execution on data after training, but we observed that classification times for all are generally negligible (although the sorting/statistic calculation steps must be performed first and these can be costly as shown in the Formatting section).
