# Supplementary material for: IntroUNET: Identifying introgressed alleles via semantic segmentation
Source: PLoS Genet. 2024 Feb 20;20(2):e1010657. doi: 10.1371/journal.pgen.1010657 (PMC10906877; doi:10.1371/journal.pgen.1010657)
Supplement: Attachment — Submitted filename: Review-PGENETICS-D-23-00137.pdf [file pgen.1010657.s022.pdf]

1 General comments

The authors present a sophisticated machine learning method to study introgression. The method goes beyond the simple identification of genomic regions exhibiting introgression as it is specifically aimed at identifying the individuals and locations in the genome that present introgressed material. The method is based on the idea of treating population genetic alignments as images that can then be analysed using Convolutional Neural Networs (CNNs). As opposed to typical applications of CNNs, that use existing data to train the network, the authors' approach generates training and testing data using population genetic simulations, a common practice in this sort of applications. A simulation study covering several scenarios of interest suggest that the method is highly accurate. In principle, the method seems sound but there are some questions about pre-processing of data and extent to which the method is of wide applicability.

As the authors state, one difficulty of applying CNNs to population genetics data is that the algorithms rely on 'spatial' information present in the images in order to carry out the classification or regression task. However, the ordering of rows in population genetic alignments is not meaningful. Following their previous contributions the authors address this problem by sorting individual haplotypes by sequence similarity. In this particular application, the authors use seriation to achieve this goal. In single-population scenarios this approach is straightforward but in the case of two or more populations, it is not as all population samples need to be ordered. Therefore, the authors start by seriating one population and then caryying out a second pre-processing step consisting in using a least-cost linear matching between the seriated and the non-seriated population. This complex pre-processing approach inevitably introduces some uncertainty as the final input image obtained will depend on the different algorithms that are used in the two steps. One also has to wonder how the choice of population for the seriation step can influence the outcome. However, the authors completely dismiss this latter concern very late in the manuscript (lines 474-478) by stating that they used the population that "**seemed** to have the most diversity to be seriated". This sounds like a very *ad hoc* approach that may work when the difference in diversity is easy to spot, which is indeed the case in the simple examples considered by the authors, but not in general. The authors should provide a more rigorous approach to carry out this step. They should also explain it much earlier in the manuscript, more precisely when they introduce the seriation step (p. 6).

Another issue that needs to be addressed is the effect of model misspecification. Although ML methods are in principle assumption-free, the training using simulated data necessarily introduces specific assumptions about the evolutionary scenario being considered. It is unclear, for example, how can training a model using data simulated under a bidirectional introgression when the real scenario consists in unidirectional introgression (or the opposite situation) affect the reliability of the results. It may be that for very well studied introgression cases, it is possible to choose the right scenario to use in the simulations, but this is not true in general. Is the proposed method really of general applicability? Either the authors need to prove that this is indeed the case or make it very clear that their method can only be applied to cases for which there is enough detailed information to make this type of decisions. The *Drosophila* example represents such a case.

2 Specific comments

1. Description of simulated scenarios: the authors should provide more details on how they simulated the bidirectional introgression scenario. Table 1 provides the distribution for the migration probability but how was this really applied? Did the authors made independent draws for each population or did they use the same value for both? In other words, was the introgression bidirectional and symmetric or bidirectional but asymmetric? If symmetric, it would be important to consider an asymmetric case.

2. Figure 3, panel A: both populations have introgressed alleles but the difference in the degree of introgression between the two populations is enormous. This highligt the need to better explain how the bidirectional migration scenario was implemented.

3. lines 254-257: You should present a figure describing this scenario in supplementary information.

4. lines 309-310: You need to provide a description of the simulation model in supplementary information. Readers should not need to search for another publication to find these details.

5. Figure 4 - "Same as (A), but for introgression from population 2 to population 1": Clarify, did you tested on data with introgression in the same direction (from 2 to 1)?

6. Lines 429-430 and 550-551: this brings about the question of whether this method is of wide applicability. What happens if you have no previous information about which regions of the genome are affected by introgression?
