# Supplementary material for: IntroUNET: Identifying introgressed alleles via semantic segmentation
Source: PLoS Genet. 2024 Feb 20;20(2):e1010657. doi: 10.1371/journal.pgen.1010657 (PMC10906877; doi:10.1371/journal.pgen.1010657)
Supplement: Attachment — Submitted filename: IntroUNET_responses.docx [file pgen.1010657.s023.docx]

Our responses to the reviewers are found below. We have also included a pdf with tracked changes, but we note that this was generated by Adobe Acrobat does sometimes, highlight a portion of text flanking changes in addition to the changes themselves, as well as a number of innocuous changes such as line number differences. We have tried to remove the latter group of highlighted differences manually but may have missed some. We hope that this is not too distracting.

**Reviewer #1:**

In this paper, the authors employ current deep-learning approaches for semantic segmentation to develop a method IntroUNET for identifying tracts of introgressed genetic variation carried within individuals under (1) a model of population divergence with a single-pulse admixture event and (2) a model of archaic introgression from an unknown "ghost" population, demonstrating improved power relative to a previous ML method ArchIE designed explicitly for that task. They also apply the method to identify introgressed haplotypes in D. sechellia, here, accounting for previously-estimated population growth in their training, validation, and testing data. They recover previously-known haplotype patterns of introgression in this species demonstrating the accuracy of their method in real application.

The paper focuses on the technical aspect of the method and is very well written. I really appreciated the explainations in the methods section. I am familiar with ML methods, but by no means an expert, and with the current text and a bit of googling, I feel like I was able to understand how the method works, what it does, and how to interperet the results.

Our response: We thank the reviewer for their kind words and are happy to hear that we succeeded in making our paper accessible (for the most part).

Reviewer 1: My comments below are about clarifying the text and assessing the performance of the method in the context of model mis-specification and realistic genomic architecture:


l 125 - 128: This sentence is a little dense. "Segmentation" needs to be defined more clearly / made more intuitive for the sake of this section. Maybe just say "In our case, we want to assign each pixel (SNP) as either "introgressed" or "not introgressed"."

Our response: We agree with the reviewer and have modified the text as follows:

“FNNs were first designed to tackle image-to-image semantic segmentation, wherein a prediction is made for each pixel in an image. Specifically, these networks take multi-channel images (usually with three color channels for red-green-blue (RGB) or hue-saturation-value (HSV)) as input and return an output of the same width and height with the output pixels denoting the type of object to which the pixel belongs [57]. Here, we adapt a FNN for semantic segmentation to classify each allele/genotype for each individual in a population genetic alignment as introgressed or not. Our approach is to treat a multi-population alignment of a genomic window as a tensor whose dimensions are *l* × *n* × *m*, where l is the number of populations in the sample (*l* = 2 in all experiments in this paper, although one could adapt this approach to examine *l* > 2 populations), n is the number of (haploid) genomes in each population, and m is the number of biallelic segregating sites in the genomic window.”

l 194 - 195: It isn't clear from the paper to what extent this architecture in Figure 2 is decided by the authors. Is UNet++ a single pre-defined architecture? Or is this one of different flavours of Unet++? Is there any transfer learning being done here?

Our response: We now clarify the definition of the UNet++ architecture, which was not pretrained, and how it differs from the original U-Net, while also specifying how our variant of the UNet++ differs from that of the original study. This text appears at the top of the “**Network architecture, training, and evaluation**” subsection of the Methods.

l 208: Maybe state directly that 'positive' means 'introgressed' and negative means 'not introgressed'.

Our response: Done.

Is the choice of weighting $w_p$ a standard way of dealing with unbalanced ratios in this approach? Otherwise, how was this decided?

Our response: Weighting examples is one of the commonly used methods for dealing with imbalanced data in machine learning, and specifically weighting according to the relative frequency of each class in the training set is one popular approach for this. We have clarified this and cited some of the relevant literature in our revised manuscript.

Also, is yHat_n the prediction probability of the positive example rather than the label?

Our response: Yes, yHat_n is the output of a softmax activation function and thus ranges from 0 to 1, which we now clarify in this portion of the manuscript.

Table 1: Easier to just say that $t_s = 4N$ generations?

Our response: Done.

In the 'simple' scenario, for bi-directional migration, is the migration rate drawn independently for the two populations? It surprised me to see so much gene flow in one but not the other direction.

Our response: The rate is drawn independently for the two directions of introgression, and we have clarified this in Table 1 and its legend.

Also, is the probability of migration the forward or backward probability?

Our response: It is a backward probability, and we now state this in Table 1’s legend.

General: How much does mis-specification of the underlying demographic model affect the performance? In particular, I'd be interested to see how the method (as trained for the 'simple' scenario in the paper) performs when the test data set has a more recent split time.

Our response: This is an excellent question and it has prompted us to examine misspecification within variants of our initial simulated scenario. Importantly, we examined not just one case of misspecification, but an entire grid of split times and maximum migration times. We trained a version of IntroUNET on each of these parameter combinations, and then tested each trained network not only on the combination it was trained under, but all other combinations as well. Although misspecification does impact performance, we were pleased to see that this effect is minimal except for the most severe deviations between the parameters of the training and test data. We describe the results of this test in a new subsection of the manuscript (**IntroUNET performs well under scenarios of model misspecification**, which also shows the impact of both direct and linked selection on IntroUNET’s performance as described later in this document), and have pasted the relevant portion below for convenience:

“Given that there may be uncertainty surrounding the population split time and also the time at which secondary contact resulting in introgression may have begun, we asked how IntroUNET’s performance was affected if these parameters were incorrectly specified during training. We generated a grid of simulated data sets for all combinations of 5 values each of the migration time upper bound and split time parameters. Specifically, the split time was set to 2, 3, 4, 5, or 6*N* generations ago, and the migration time upper bound, expressed as a fraction of the split time, was set to 0.1, 0.2, 0.3, 0.4, or 0.5. This yielded a total of 25 parameter combinations, and a training and test set was generated for each. A version of IntroUNET was then trained on each of these training sets, and each trained network was then tested on all 25 test sets. As shown in Figure S2, accuracy was acceptable in most cases of misspecification, and misspecification of the split time had only a modest impact on performance. Interestingly, accuracy suffered the most when the network was trained on a wide range of introgression times but only recent introgression had occurred. On the other hand, when trained only on more recent migration times, accuracy on data with a wider range of migration times remained high in most cases. This may imply that training on the lower end of one’s confidence window of the migration times could be a reasonable strategy, but repeating this analysis on a wider range of demographic models may be required to confirm the effectiveness of this approach.”

l 294 - 297: Was the (not-downsampled) training data set the same for ArchIE as for IntroUNET?

Our response: Yes, it was the same data set, and we have clarified this in the manuscript.

l 310: It might be more accurate to say "Following [39], we used dadi to estimate..."

Our response: Fixed.

Between lines 323 and 324:
i) Were all 100 bootstraps used for sims, or only those listed in supp table 1?

Our response: Of the 100 bootstrap site frequency spectra that we used as input to dadi, we first filtered out those that did not have at least 5 successful optimization runs from different starting points. A total of 57 replicates survived this filtering step. We then filtered another 14 estimates that had much lower log-likelihoods than the authors, leaving 43 parameter combinations that we used to simulate our training data. We have clarified this in the Methods and the table legend.

ii) With regard to estimating continuous migration but using a pulse-admixture for training, validation, and testing... This is a bit funny to me. Those parameters estimated by dadi are all correlated with each other.

By taking those values out of the continuous-migration context and using them in a pulse-migration context, how well do they still represent the population under study? I think this is a good point for a model-misspecification test. How well does the network trained on pulse-admixture perform when tested on data simulated under the IM model?

Our response: It is true that because these parameters are jointly estimated, they are correlated with each other and we understand that it may seem odd to remove some from the model. Our reason for not directly using the same model estimated by dadi is one of convenience: our inference pipeline (modified from Schrider et al. 2018) produces commands for variants of the ms simulator, which we could only use to track introgressed alleles in pulse-migration scenarios. We considered porting these models to SLiM, but this would potentially result in very long run times (and might necessitate an investigation of the impact of rescaling on signatures of introgression and efforts to find them).

However, we do not feel that the discordance between the continuous migration model inferred by dadi and the pulse-migration events used in our training simulations is a major concern. Our rationale for this is simply that the parameters estimated by dadi refer to a genome-wide demographic model, but IntroUNET is not trained to make inferences on genomes, but rather small segments of genomes. If continuous migration has been acting within a population at relatively low rates, and we note that the rates are low in the *Drosophila* data set examined here, then we would expect that most regions that have experienced introgression experienced only a single event, and our examination of the putative introgressed regions in the *D. sechellia* genome supports this. For example, the regions shown in Figure S15 seem to be consistent with a single introgression even within each window (although some introgressed haplotypes may be shorter because of historical recombination events).

Thus, we do not feel that training our method on instantaneous migration events is inappropriate, so long as the method can handle the possibility that different genomic regions have experienced introgression events at different times. For this reason, our training simulations include a range of possible migration times. The only concern from our perspective is therefore the possibility that the timing of migration events may differ between the training data and the real data, and we feel that this problem is addressed sufficiently in our response to the reviewer’s earlier misspecification comment. Although we do not feel that an additional analysis of misspecification is required for this, we do feel that it is worth alerting this issue to readers, and we therefore have added the following text to the Discussion:

“…estimated continuous migration parameters between *D. simulans* and *D. sechellia*, but we trained IntroUNET using a modified version ms that can only track introgressed alleles resulting from pulse migration events. Because introgression in these data appears to be relatively rare, affecting only a minority of the genome [56, 39], those loci that are affected typically will only have experienced a single introgression event—-note that the example regions in Figure S15 are consistent with this. Because IntroUNET is trained on simulations that experience only a single introgression time within a given region, but the migration time varies across regions, this form of misspecification is a problem only inasmuch as it may result in migration times differing between the true and simulated data. Thankfully, our results in Figure S4 suggest that IntroUNET will typically perform quite well in the presence of such misspecification. In addition, we note that IntroUNET can be used on unphased data and experiences only a small decrease in performance relative to the phased case (Figure S10). This property, combined with its robustness to misspecification, make it a versatile tool that can be applied to model and non-model systems alike.”

Also, just out of curiosity, would it be possible to use the dadi-inferred IM demography directly for training purposes?

Our response: See above.

iii) label smoothing: This may be a standard thing for deep learning approaches, but it was confusing for me as a PGen reader. You tell me what it does, but I don't have any intuition as to why it helps.

Our response: We now share a reference to label smoothing that curious readers can consult if they wish to learn more about label smoothing (<https://arxiv.org/abs/2006.11653>).

l 326: It wasn't clear how this is helpful for training or what problems this helped to resolve. Is there a reason this and the Platt scaling are only used in the Drosophila analysis?

Our response: We have clarified this in the Methods as follows:

“Neural networks are often found to be poorly calibrated or biased in their predictions resulting in overconfidence or underconfidence in the posterior probabilities, even if they achieve high classification accuracy (Guo et al. 2017). Platt scaling simply finds a line of best fit (a slope and intercept) for the predicted logistic scores given by a pre-trained network and the ground truth via the unweighted cross entropy function. We observed poor calibration in our test simulations for this *Drosophila* model. Thus, After training, we then learned a posterior probability correction via Platt scaling (Plat et al. 1999), which we found to produce better calibrated estimates of the probability of introgression than the raw output from IntroUNET. The recalibrator was trained via gradient descent for 25 epochs on the unweighted cross entropy loss calculated on the validation set. This was accomplished via PyTorch and the code to do so is also included in the IntroUNET GitHub repository. We note that Platt scaling was used in both on our simulated evaluation set and when applying it to real data below, giving us better confidence in the posterior probabilities computed in our analysis of predicted introgressed haplotypes in \dsech. An example input along with true and inferred outputs of the *Drosophila* simulated scenario is shown in Figure 3C. We did not compute Platt corrections for the other problems examined in this paper, as we did not examine posterior probabilities for further analysis, and classification accuracy was adequate without this scaling. We also note that ROC and precision-recall curves are not expected to be impacted by recalibration.”

l 339-340: Do you have any intuition about why this happens?

Our response: Although we do not know the cause of this for certain, it is possibly due to the very low level of diversity in *D. sechellia* genomes. If a false positive introgression call is made, there is a chance that it will be repeated across *D. sechellia* genomes because of their high degree of similarity. We now share this potential explanation in this part of the Methods, while appropriately noting its speculative nature.

l 391 - 392: What classification threshold was used in the manuscript? 0.5?

Our response: That is correct, and this has been clarified in the Methods.

l 451 - 453: I may have misinterpereted this, but the frequencies of the introgressed haplotypes in the case of positive selection doesn't seem very high relative to the background. Aos, the fact that there's a U-shape to the distribution seems more important than the mean. Do you have any further insight or intuition about this?

Our response: Absolutely! We feel that this U-shaped distribution is highly consistent with the expected pattern some distance away from a sweeping introgressed allele, as linked neutral introgressed alleles should hitchhike to high frequency in relatively close proximity to the target of selection, and intermediate frequencies at somewhat further distances. This is shown quite nicely in Figure A.3 from Setter et al. (<https://doi.org/10.1371/journal.pgen.1008867>). We now make this argument at the bottom of the relevant section of the manuscript (**IntroUNET accurately detects introgressed haplotypes between *D. simulans* and *D. sechellia***).

l 479: As a reader, I want to know how feasible it is for me to run either of these methods given the resources I have access to. A supp table comparing the computation costs for archie and introUnet at the various stages would make this more clear.It would alos help to indicate the stages that can be parallelized in the respective approaches.

Our response: This is a helpful suggestion! We have added Table S2 to the manuscript, which addresses this concern.

l 514 to 515: It isn't clear to me how much the sample size and number of snps (input image size) affects the cost to train the model.

Our response: Computational speed scales approximately linearly as any dimension of the input increases in size. We observed the same as the number of training examples increases. We now note this in a new paragraph at the end of **IntroUNET accurately identifies introgressed alleles in a simulated dataset**. In this paragraph, we also examine the impact of each of these three properties on classification performance, by visualizing the trajectories of the loss function during training (Figure S3).

l 546: With these image-processing ML methods, I tend to wonder how much the underlying genome architecture affects performance, and I think this could be investigated directly in this paper. I would like to see a small set of test simulations run in SLiM using the same population parameters here but including the D melangoaster genome annotation, genetic map, and distributions of deleterious fitness effects (they are in the standard popsim catalogue). Testing this data with the naive model would also help demonstrate the robustness of the method in real applications.

Our response: This is a great suggestion, and we have followed the reviewer’s advice and implemented this model. The results are described in the last paragraph of our new section on model misspecification (**IntroUNET performs well under scenarios of model misspecification**). We are highly encouraged by IntroUNET’s performance in this scenario: although there is a notable increase in the false negative rate, our false positive rate is actually decreased slightly, suggesting that the introgressed regions that we infer from real genomes should be accurate in spite of unmodeled background selection.

l 549: What types of advances would help resolve these issues? More extensive training with a broader range of parameters? Improvements to machine learning techniques themselves?

Our response: As we now state in the Discussion, it may be that the very low level of diversity in the *D. sechellia* population is contributing to the false positive introgressed segments shared by the entire *sechellia* sample. It is possible that a large increase in the size of the training data (the number of examples and/or the window size) could help with this, and could also reduce the false positive rate enough so that an initial window-based classification step is no longer required, but having not experimented with this extensively, we have opted not to include such speculation. However, we have included a window-based classifier, similar in spirit to the network used by Flagel et al. 2019 (<https://academic.oup.com/mbe/article/36/2/220/5229930>), with the IntroUNET package, which will allow users to run this initial step if necessary without downloading/running any additional software. This classifier is described in a new subsection in the Methods (**Window-based classifier for detecting introgression**), and its performance is described in the Discussion. As shown in Figure S18, this classifier identifies introgressed regions with excellent accuracy.

l 574-576: What experimentation did the authors do to decide on the architecture in figure 2? How sensitive is the method to the choice of architecture? If this matters, then how feasible is it for an IntroUNET user to find the optimal architecture and hyperparameters?

Our response: We did not experiment with different types of architectures, opting initially for the UNet++ style of architecture given its strong performance in image segmentation. Regarding hyperparameter tuning for this architecture, we have added the following text to the Methods which states what we did (and did not) do:

“Although we did not conduct systematic hyperparameter optimization for this architecture, we did experiment some with different hyperparameter values and in general found that large networks (i.e. networks with more layers or more learnable parameters) performed better than smaller and more shallow networks. However, in general we found that, for this particular network architecture, the choice of hyper-parameters had a limited impact on performance. We therefore applied this architecture to each problem considered in the paper.”

**Reviewer #2:**

Ray and collaborators present a deep learning algorithm for semantic segmentation adapted to identify introgressed alleles in genome alignments. This work aligns with the mounting amount of studies on applying deep learning in genomics, highlighting its potential to provide evolutionary inferences in the genomic era.

From our point of view, this work’s most significant and original contribution is using semantic segmentation (based on an adapted version of UNET++) to tackle the problem of identifying introgressed alleles/haplotypes in an alignment of sequences. Compared to local ancestry inferences, significant improvements of IntroUNET are that a reference panel of donor populations is not required, and the introgression process is explicitly modelled in the simulations (although parameter values of the demographic scenario are not inferred). Moreover, it extracts genealogical information directly from the raw alignment of sequences by passing the summary statistics step, which a loss of information can accompany.

To say a word about the form, the writing style is generally clear with a very nice Introduction, the figures are well done, and the manuscript is pleasant to read. However, it is too technical in some places at the expense of biology, and some assumptions are not clearly stated, making the application of the method to actual data not so easy. Still, we are convinced that such a tool could bring numerous benefits to the field of population genomics. Below are a series of comments that we hope will improve the manuscript.

Our response: We thank the reviewer for their time and extensive feedback on our manuscript. We have conducted a great deal of additional analyses and made extensive revisions to our paper to address them. These are detailed in our responses below, but we note that, because there was a fair amount of overlap in some of the reviewer’s concerns and we wished to avoid redundancy in our responses, in certain instances our responses are somewhat brief because they refer to more detailed responses to reviewer 1 that address the same concern.

1. Scenarios

● In general, we are a little afraid that the scenarios modelled by the authors to test their IntroUNET method are a bit too simple to evaluate its ability to correctly identify introgressed haplotypes with actual empirical data. Indeed, in the first scenario, which is on purpose a simple case of introgression, the authors model a single pulse of admixture occurring recently (between the present and ¼ of the split time) with a fraction of individuals introgressed comprised between 10% and 50% (thus setting aside cases where introgression is rare, as expected around species barriers for example). This configuration is favourable for the detection of introgressed haplotypes, all the more so as population sizes are small, which faster the incomplete lineage sorting and reduces the risk of confounding it with introgression. We are convinced that testing the method against other scenarios, such as ancient migration or a scenario with continuous migration (not just admixture), would be more useful.

Our response: We appreciate the reviewers’ concern that our method needed to be tested on more challenging scenarios, and are very pleased with IntroUNET’s performance on these tests. For example, we have now greatly expanded the number of scenarios that IntroUNET has been applied to, including a large grid of introgression and split timing parameters. Importantly, we find that misspecification of these parameters during IntroUNET’s training has only a minimal impact on accuracy. These results are detailed in the second paragraph of a new subsection of the Results, entitled “**IntroUNET performs well under scenarios of model misspecification**”. Additional test cases of misspecification (involving natural selection) are detailed in response to your next comment:

● We were surprised the authors used a forward-in-time simulator (SLiM) to simulate the first scenario, which is purely neutral as the other two scenarios. A coalescent approach seems more suited to this kind of simulation. By using SLiM, we would have expected the authors to explicitly include selection in their forward simulations (e.g. adaptive introgression or the presence of incompatible loci between species), which would have been useful to compare with their analysis of the Drosophila data. Another possibility to capture the effect of (linked) selection is to simulate under the coalescent and allow for heterogeneity in model parameters such as introgression rates or effective sizes (e.g. Roux et al. 2016 https://doi.org/10.1371/journal.pbio.2000234). This might not capture well the effect of adaptive introgression. Still, background selection and selection against migrants/hybrids can be well captured in that way, and these are also important widespread processes. In particular, it would be useful to assess how heterogeneity in incomplete lineage sorting across the genome (by varying Ne across loci in the simulations) affect the inference of introgressed tracts by IntroUNET. And more generally, it is important to delineate the conditions under which the method works correctly or not.

Our response: We apologize for any confusion caused by our use of a forward simulator for some of our simulations. This was necessary because the modified version of the coalescent simulator ms that we used did not properly track introgressed haplotypes in the case of bidirectional introgression. Nonetheless, as outlined in our responses to Reviewer 1, we have now expanded our SLiM simulations to generate test sets incorporating the effects of natural selection. These include cases of direct selection both for and against introgressed haplotypes (described in the third paragraph of the **IntroUNET performs well under scenarios of model misspecification** subsection). We also used SLiM to examine the impact of background selection, incorporating gene annotations, estimates of the distribution of deleterious fitness effects, and the recombination rate landscape, all estimated from *Drosophila melanogaster*. These results are also detailed in the subsection **IntroUNET performs well under scenarios of model misspecification** (final paragraph). As you may gather from the title to this subsection, we were happy to see that IntroUNET performs very well in the presence of these forms of training model misspecification.

● The ghost introgression scenario is presented in Figure 3 as an ‘Archaic human ghost introgression scenario’. However, there are no biological justifications for the parameters chosen here. The authors refer to the study by Durvasula, which seems insufficient. Furthermore, the two methods (IntroUNET and ArchIE) require a reference non-introgressed population to test for ghost introgression in the focal population. However, it could be tough to know a priori which population is suitable as a reference in the case of ghost introgression because the source lineage is typically unknown. Methods without references are more appropriate in that situation (e.g. Chen et al. 2020 https://doi.org/10.1016/j.cell.2020.01.012). So the authors should clarify that IntroUNET is most suited for cases where the source population is suspected but unsampled or extinct (not unknown).

Our response: We now state in the Results, when discussing the model from Durvasula et al., that this model was motivated by the task of identifying Neanderthal-to-human introgression. In addition, we now state in the Discussion that in some cases a non-introgressed reference may not be available, and cite several methods that could be used in that scenario (including Chen et al. 2020).

2. Applying to real data

● The authors clearly show that their method can identify introgressed alleles/haplotypes in simple demographic scenarios. However, when one goes to more complex (and so realistic) scenarios, IntroUNET seems to face some difficulties. Notably, the authors indicate along L334 that the Drosophila version of IntroUNET performs best when some introgression is present in the input. Therefore, they present results on regions previously found to contain introgression in the required direction (i.e. from D. simulans to D. sechellia).
The first issue is that most users of the method won’t know a priori the regions that introgress between their study species. Therefore, one should probably expect poorer evaluation results than those presented in Figure 6.

Our response: We agree that this was an important limitation, and have therefore sought to address it by including in the IntroUNET package a window-based classifier for identifying genomic regions that have experienced introgression, along with the direction of introgression. This method is similar in spirit to the network used by Flagel et al. 2019 (<https://academic.oup.com/mbe/article/36/2/220/5229930>) to detect introgression, and we show in Figure S18 that this method is very accurate. We describe this Method and its implications in the Discussion. Specifically, we state that:

“To improve the usability of our method, we have therefore included in the IntroUNET package a neural network for classifying genomic windows as having experienced introgression or not, in the same vein as the approach taken by Flagel et al.. As shown in Figure S18, when trained to detect genomic windows that have experienced introgression between *D. simulans* and *D. sechellia*, is highly accurate. We recommend that users test the performance of IntroUNET on simulated data prior to analyzing real data, and if they observe unsatisfactory performance in non-introgressed regions in simulated test data, running our window-based classifier as a first step may allow users to proceed with accurate segmentation on regions that appear to have experienced introgression, as we were able to do for our *Drosophila* dataset.”

The second issue is that this version of IntroUNET occasionally produces many false positives. And the way false positives are handled seems not satisfactory. Indeed the authors simply discarded sites that were predicted to be introgressed in more than half of the recipient genomes, considering these are artefacts of their predictions. This may be a suitable method in situations where introgression events are known a priori, as in this Drosophila case. But it can never be a general solution as, in most situations, we won’t be able to disentangle artefacts from biological signals, i.e. the actual number of true positive targets isn’t typically known. This is an issue as the article aims to present a new general tool. These caveats on the applicability of the method should be spelt out.

Our response: This is a valid point that we feel is addressed by the inclusion of the window-based classifier noted above into the IntroUNET repository. This allows users to train both this classifier and the IntroUNET segmenter on the same dataset, minimizing the additional overhead required to run both methods sequentially.

● In the Drosophila scenario, the demographic parameters were first estimated with ∂a∂i under a model of isolation-with-migration and exponential population size change. However, the simulations were performed using single-pulse introgression, which isn’t justified. If the idea was to show the identification of the introgressed haplotypes is robust to deviations from the simulated scenario by IntroUNET, this should be highlighted.

Our response: This is a fair point that was also raised by Reviewer 1, and we therefore discuss this more at length above (see the comment numbered with “ii)” by Reviewer 1). In short, if the rate of introgression is not extremely high, a genome-wide continuous migration rate model should typically produce between zero and one introgression events at individual loci. Thus, a pulse-migration model is adequate, so long as the method is trained to handle variation in these migration times across loci (which IntroUNET is, thanks to the variation in these times across training examples). However, if the true model is a continuous migration model estimated from data, and the training model is a pulse migration model with times varying from locus to locus, as is the case here, then there may be some discordance between the distribution of introgression times between the real data and the training data. Thankfully, this is just the type of misspecification that we address in “**IntroUNET performs well under scenarios of model misspecification**” where we show that our method performs quite well in this case.

● We think that Figure 7, which shows the distributions of introgressed allele frequencies, doesn’t really convey well the results the authors want to highlight. To help see the difference between categories (panel A: genic vs intergenic, panel B: sweep vs outside the sweep), the authors could draw vertical lines showing the average of each category (as reported in the text). Nevertheless, the differences in the average introgressed frequencies are quite small (3.2 vs 3.7 in the two comparisons), providing weak evidence that introgression is stronger in intergenic rather than genetic regions or stronger in the sweep region. It would be more informative, at least for the adaptive introgression case (panel B), to plot the length of the introgressed haplotypes as a function of their frequency, as long coincident haplotypes are expected around positively selected sites (as shown in Supp. Figure 5).

Our response: Excellent suggestion! Haplotype lengths are indeed very informative here, and we have therefore examined how IntroUNET’s predicted haplotype lengths increase at increasing proximity to the adaptive introgression region on chr3R. The results are quite striking, with a nearly 12-fold increase in haplotype lengths closest to the sweep compared to those 100 kb away. We now share this finding in the Results and in Figure S16.

As a note, it is a bit surprising that the adaptively introgressed alleles are not fixed in the recipient population: is that because the sweep is ongoing?

Our response: We focused our analysis on regions linked to the sweep but not directly in the sweep’s epicenter, with our rationale being that at these further distances from the target of selection enough diversity has survived the sweep so that introgressed haplotypes and IntroUNET’s predictions can be more clearly visualized. We have clarified this in the text, stating that “we show IntroUNET's predictions for three example windows near the sweep, but far enough away to contain appreciable amounts of diversity.”

● The dimension of the input data in the simulations seems quite small (100 Kb regions analysed in windows of 125 polymorphisms) if the idea is to use IntroUNET to scan genomes for introgressed alleles/haplotypes. It would be helpful to specify in the 4.5 section the computational time (and under which multithread setting) when scanning for genomes of, say 500 Mb or 1 Gb.

Our response: As detailed in our response to Reviewer 1, we have included more details on IntroUNET’s computational cost and how its performance improves with the input size in Table S2 and Figure S3, respectively. Also, as noted in the second paragraph of **IntroUNET accurately identifies introgressed alleles in a simulated dataset**, as the input size increases the running time seems to increase linearly, but GPU memory requirements also increase.

● Another feature of the data that is not clearly spelt out is that haplotypes are required. This is important to specify early in the manuscript as potential users need to know which data type suits the method. Typically, it is still not common to get phased data (or have at its disposal inbred lines). So this limits the applicability of the IntroUNET method as it is presented and tested here (i.e. against haplotypes).

Our response: This is another great point. We have added a section to the manuscript entitled “**IntroUNET can handle unphased data with minimal loss of accuracy**” where we describe a version of IntroUNET that has been modified to take diploid genotype matrices as input, and is able to infer which individuals have experienced introgression with nearly the same accuracy as obtained by the original version.


3. IntroUNET

● This deep learning algorithm for semantic segmentation allows classifying alleles into two classes: introgressed or non-introgressed. However, it is not designed to infer the best parameter values of the demographic scenario modelled, such as the time of admixture. We suggest the authors spell out this is not the aim of their method, as it could be confusing for readers unfamiliar with such a classification method.

Our response: We have rephrased a sentence in the Introduction that will now make this clearer: “Here, we describe IntroUNET, a fully convolutional neural network that examines a two-population alignment and seeks to identify which alleles in each individual were introgressed from the other population.”

● Some choices are not precisely justified. For example, it is unclear why the authors used SLiM in the first scenario (simple bidirectional introgression) while they used “msmodified” in the second (ghost introgression) and third (introgression in Drosophila) scenarios.

Our response: We have addressed this above, but also now state in the manuscript (“**Training a U-Net to detect ghost introgression**”, where msmodified is first mentioned) that msmodified can only be used to track unidirectional introgression.

● The authors provide in Figure 3 the typical outputs of IntroUNET. But it is unclear how ones go from the matrix of the inferred introgression probabilities to the matrix of the inferred introgressed haplotypes (i.e. the binary classification). Did the authors apply an arbitrary probability threshold to classify each allele (e.g. if prob>0.5, then the “introgressed class” is depicted)?

Our response: Yes, if IntroUNET’s predicted introgression probability is >0.5, then we count that as a predicted introgression event, and this has been clarified in the manuscript as described above.

● The authors mention along L210-2016 that the imbalance in the data (i.e. the fact the “introgressed” class is much less frequent than the “non-introgressed” class) may affect evaluation metrics. However, it is not clear from the text to what extent the configuration of the weighted form of cross-entropy affects the predictions made by the network, e.g. if the data is heavily imbalanced. Could you please explain more clearly what has been done to handle this issue?

Our response: In light of this comment and a similar comment from reviewer 1, we have expanded and improved our explanation of the class weights in this portion of the Methods, while citing relevant literature. Moreover, we stress that the accuracy values reported in our confusion matrices are all balanced (i.e. obtained by taking the average of the percentages along the diagonal elements), so the class imbalances have no impact on this metric, and this is also noted in this portion of the Methods.

● In the next paragraph (L217 to 226), the authors mention some technical constraints associated with the input image (the dimensions must be multiple of 16). Therefore, a step of individual up-sampling has to be applied. Again, the implication of such handling is not discussed in the manuscript.

Our response: We now state in this portion of the manuscript that this may cause a slight decrease in the variance of predictions made for those individuals arbitrarily chosen to be upsampled (since their final prediction is the mean of multiple predictions). Given IntroUNET’s performance, this does not appear to be a major issue, but it is still one worth noting as we have now done.

● Nor discussed is the difference between IntroUNET and ArchIE regarding inputs: the first focuses on polymorphic sites, while the second includes invariants. Is that important to include invariants for the classification method, or does it just burden the simulations?

Our response: In our experience, incorporating positional information (which would tell the network about the number of monomorphic sites between each pair of polymorphisms), adds very little accuracy to inferences. However, this is not something that we have experimented with systematically for IntroUNET. We therefore now note this in the manuscript (final sentence of the **Method overview** subsection).

● Evaluation metrics are calculated globally, considering all genomic windows under study. However, ones can expect the ability of the method to identify introgressed haplotypes to depend on local features along the genome, e.g. local recombination rates. It would be useful to assess its effect by running a different version of the first scenario where the recombination rate is a magnitude lower than the mutation rate.

Our response: This is an excellent suggestion, and as described above we have now incorporated simulations demonstrating that IntroUNET remains able to detect introgression with high precision in the presence of recombination rate variation and background selection. As shown in Figure S9, IntroUNET’s performance does not suffer at lower recombination rates.

4. Technicalities

As a general note, specific technical aspects in the manuscript would not need to be presented in the article's main body considering the expected readers of this journal. We suggest adding a Supplementary Information section with the deep learning details (e.g. the learning rate, the actual loss function used, the potential issues of class imbalance and ordering individuals, etc.). This change will make room for important points that need to be explained in the current version (as the reader is currently expected to have some basics of deep learning, which won’t probably be the case).

Our response: Because this paper is primarily focused on the presentation of a new computational method, we have opted to keep our now-expanded explanation of our deep learning approach in the main text of the paper so that readers do not have to open multiple documents to see the full picture. However, if the editors feel that this information should be moved to a supplemental section, we would heed their advice.

● The « 3.1.1 Ordering individuals within the input image » section is presented a bit too early. We suggest moving it after the current part 3.1.2, which covers the network architecture, training and evaluation. We also suggest reducing it significantly in the main body and leaving the details for the Supp. Info. We acknowledge that resolving the seriation problem is very important when using this type of deep learning (and it illustrates that convolutional features are not perfectly suited for images such as genomic alignments). Still, it appears too detailed for the intended public of the journal (which, for the most part, will have a good background in genetics but no advanced knowledge in mathematics or computer sciences).

Our response: We appreciate this section, but upon reflection we feel that it makes more sense to first give a description of the input to the network (i.e. what it is looking at) before describing the architecture of the network (how it is looking at it). However, we again reiterate our offer to reorder things and create a new supplementary section if requested by the editors.

● The « 3.1.2 Network architecture, training, and evaluation » section lacks important information, in our opinion. Going over some basics of deep learning, especially regarding training and evaluation of the method, would be helpful for the most inexperienced readers. In addition, it would be difficult for a reader unfamiliar with deep learning methods to understand Figure 2: it is unclear what numbers under brackets are, why they change through the convolutional network, and why there are two matrices in the output. These points should be clarified in the legend. Moreover, an effort must be made to explain to non-experts the IntroUNet architecture described from L195 to L204. On L225, please, explain what a batch size is.

Our response: Thank you for the suggestion! We have greatly expanded our explanation in **Overview of neural network optimization** to clarify each of these things.

● Precision, recall, AUPR and AUROC curves are expected to be already known notions for the readers. It might be good to give short reminders as to what each of these metrics represents. Stating that these metrics evaluate the network's ability to make correct predictions without giving some reminders of what they mean is unsatisfying. For example, the PR curve for the “ghost introgression” scenario is worse than the one for the previous scenario, and the shape of the curve contains interesting information that the reader should understand.

Our response: We have added explanations for these curves where they are first mentioned, which is in Methods when discussing data imbalance, as follows:

“Note that this data imbalance can affect evaluation metrics such as the ROC curve, which plots a method's true positive rate against its false positive rate, thereby showing how much specificity must be relaxed (by decreasing the detection threshold) to achieve a given sensitivity. For this reason we also compute precision-recall (which shows how a method's positive predictive value decreases as its sensitivity increases) which is known to be robust to unbalanced binary inference problems as it does not incorporate correctly predicted absences (Sofaer et al. 2019).”

● The use of Platt scaling seems to be helpful considering the improvement in accuracy (Figure 6), but once again, it is hard to understand this procedure without prior knowledge. We suggest adding information about Platt scaling in the Supp. Info. Furthermore, according to Supp. Figure 4, it seems that IntroUNET (before scaling) tends to consistently overestimate the introgression probability. Could you please comment on that?

Our response: Yes, we do state in the Results that the uncalibrated version of IntroUNET was producing inflated introgression probabilities. We also now explain Platt scaling as follows:

“Neural networks are often found to be poorly calibrated or biased in their predictions resulting in overconfidence or underconfidence in the posterior probabilities, even if they achieve high classification accuracy (Guo et al. 2017). Platt scaling simply finds a line of best fit (a slope and intercept) for the predicted logistic scores given by a pre-trained network and the ground truth via the unweighted cross entropy function. We observed poor calibration in our test simulations for this *Drosophila* model. Thus, After training, we then learned a posterior probability correction via Platt scaling (Plat et al. 1999), which we found to produce better calibrated estimates of the probability of introgression than the raw output from IntroUNET.”

5. Minor comments

● Section 3.2.1: as we understand, populations A and B are drawn from the same set of parameters. If that’s the case, what is the point of studying both the introgression from A to B and B to A (see Figure 4)? Is that for showing IntroUNET can correctly detect the introgression rate in each direction if it is asymmetric? Please, clarify this point.

Our response: We have clarified this in Results as follows: “The examination of the two unidirectional cases is a useful sanity check, as the two populations experience identical demographic histories and thus performance should be similar in both cases, which is indeed the case.”

Moreover, it is unclear from the text that the fraction of individuals introgressed can vary freely in each direction (i.e. it can be asymmetric); please clarify this.

Our response: Yes, this fraction does indeed vary freely in each direction. We have clarified this in the manuscript as described in our response to reviewer 1.

Finally, in Table 1 (and also in Table 2) it would be more precise to replace “Migration time” with “Time of introgression event”, and “Migration probability” with “Fraction of individuals introgressed”.

Our response: We have made these changes and further simplified and clarified these tables (as well as correcting some confusing information in Table 2 that stemmed from an initial run of this model in SLiM that was replaced with the msmodified simulations for improved computational speed).

● Sections 3.2.2 & 3.2.3: the authors do not specify the specific parameters used to train their network in IntroUNET. Please specify what the numbers of epochs are, the patience, etc.

Our response: We now state in the Methods: “For all problems considered we trained the network for a maximum of 100 epochs, with a patience of 10 epochs (meaning we halted training if for ten consecutive epochs validation failed to decrease below the previous minimum).”

● Section 3.2.3: Label smoothing is used in the Drosophila dataset to add some noise to the labels, and it seems to help with the training, as stated by the authors on L326. But this is a bit vague and would require further explanations.

Our response: We have added the following: “Label smoothing regularization has been shown empirically and theoretically to reduce the variance of the noise in estimating the gradient for optimization problems when using stochastic gradient descent (Xu et al. 2020).”

● Section 4.3: on L416, it is unclear what are these “43 inferred demographic models” since a single scenario of isolation-with-migration was modelled with ∂a∂i. Please, clarify if they correspond to different multi-parameter estimates under the same demographic model. From L436 on, it would be more meaningful for readers if the numbers of introgressed haplotypes were transformed into fractions.

Our response: We apologize for the confusion here. We have clarified this section as described in our response to reviewer 1 (see third paragraph of “**Application to real data: finding introgressed regions in *D. simulans* and *D. sechellia***”).

Furthermore, the simulation setting (i.e. the recombination rate, the mutation rate, etc.) used in the Drosophila case is not stated in the methods.

Our response: Thank you for catching this omission! These parameters have been added to this same section of the Methods.

● Section 4.5: this kind of comparison is useful, but it would be clearer to present it in a table. Moreover, some more « general guidelines » as to how long the execution of this pipeline would take, considering other concrete examples, would be a welcomed addition. The indication on L574 that using IntroUNET may involve experimentation to identify the optimal network architecture should perhaps also be included in the computational costs calculated in Section 4.5.

Our response: We have added this information in the form of Table S2 to the manuscript. We do note that hyperparameter optimization would increase these costs, but we also note in **Network architecture, training, and evaluation** that the minimal hyperparameter tuning that we did perform during the analyses for this paper did not have a large effect on accuracy.

6. Typos & formatting

● L271: “as we described in 1”, please explain what “1” refers to.

● L343: typo in “recorded [the] the fraction (...)”.

● L343: “to have experienced introgressed at that site” seems a repeat of the start of the sentence.

● L367: “Reference-free inference of archaic local ancestry”: the title is confusing as the second scenario (archaic introgression) requires a target population and a reference un-introgressed population. Please, clarify.

● L376: typo in “Again, we trained our [ ] and tested our (…)”.

● L455: brackets are missing for “Supplementary Figure 5”.

● L572: typo in “a high-performance compute[] cluster (…)”.

● In the discussion, at several places, a space is missing after the species name D. simulans and D. sechellia.

● Legend of Table 1: “After some amount of time of complete isolation, which follows the described uniform distribution”. Isn’t the split time fixed in the simulations (ts=4N with N=500)?

● Legend of Figure 3: replacing “split followed by recent gene flow” with “split followed by a single-pulse introgression event” would be more precise.

● Legend of Figure 5: the legend does not indicate panels C and D.

● Supp. Figure 1: please, scale the introgression probability panels to that of the other panels.

● Supp. Figure 2: although it is stated that two examples without introgression are shown for each method, this seems not to be the case for Archie, as three examples have nearly zero introgressed haplotypes in the true panels.

● Legend of Supp. Figure 2: typo in “Each [ ] shows the true and (...)”.

● Supp. Figure 5: please, provide the unit of the x-axes.

Our response: All fixed!

**Reviewer 3:**

1 General comments

The authors present a sophisticated machine learning method to study introgression. The method goes beyond the simple identification of genomic regions exhibiting introgression as it is specifically aimed at identifying the individuals and locations in the genome that present introgressed material. The method is based on the idea of treating population genetic alignments as images that can then be analysed using Convolutional Neural Networs (CNNs). As opposed to typical applications of CNNs, that use existing data to train the network, the authors’ approach generates training and testing data using population genetic simulations, a common practice in this sort of applications. A simulation study covering several scenarios of interest suggest that the method is highly accurate. In principle, the method seems sound but there are some questions about pre-processing of data and extent to which the method is of wide applicability.

As the authors state, one difficulty of applying CNNs to population genetics data is that the algorithms rely on ’spatial’ information present in the images in order to carry out the classification or regression task. However, the ordering of rows in population genetic alignments is not meaningful. Following their previous contributions the authors address this problem by sorting individual haplotypes by sequence similarity. In this particular application, the authors use seriation to achieve this goal. In single-population scenarios this approach is straightforward but in the case of two or more populations, it is not as all population samples need to be ordered. Therefore, the authors start by seriating one population and then carrying out a second pre-processing step consisting in using a least-cost linear matching between the seriated and the non-seriated population. This complex pre-processing approach inevitably introduces some uncertainty as the final input image obtained will depend on the different algorithms that are used in the two steps.

Our response: We thank the reviewer for their time and attention to this manuscript. Again, there is a fair amount of overlap between some of these comments and those of the previous reviewers, and in those instances we may give a brief summary while referring to a more detailed response shared above.

Thank you for sharing these thoughts on our preprocessing steps. We of course agree that these steps are consequential, which is why we have included a section in the manuscript examining their impact (**Sorting via seriation improves introUNET's accuracy**). To ensure that readers understand the implications of these steps, we now preface this section with the following statements:

“As described in the Methods, IntroUNET preprocesses its input by first ordering the individuals of one population and then matching individuals in the second population to those in the first, and the choice of algorithms used for these steps (or whether to perform them at all) will therefore influence IntroUNET's output.”

However, given that we have now shown our method to perform accurately on a variety of additional simulated scenarios (including misspecification of population split times, misspecification of introgression event times, the presence of unmodeled positive or negative selection acting introgressed haplotypes, and the presence of unmodeled background selection and recombination rate variation, all described in the new section entitled **IntroUNET performs well under scenarios of model misspecification**), we our confident that our method is still of general utility in spite of these preprocessing steps (which we show increase accuracy).

One also has to wonder how the choice of population for the seriation step can influence the outcome. However, the authors completely dismiss this latter concern very late in the manuscript (lines 474-478) by stating that they used the population that ”seemed to have the most diversity to be seriated”. This sounds like a very ad hoc approach that may work when the difference in diversity is easy to spot, which is indeed the case in the simple examples considered by the authors, but not in general. The authors should provide a more rigorous approach to carry out this step. They should also explain it much earlier in the manuscript, more precisely when they introduce the seriation step (p. 6).

Our response: We have considered the reviewer’s point here but do not feel that this is a major issue. If the two populations differ substantially with respect to their level of diversity, as is the case in our *Drosophila* example, then this choice is easy to make. If instead that populations have diversity levels that are very similar or even identical (as is the case in our first simulated example scenario), then the choice of which population to sort first will have no impact on performance. Importantly, we have shown that IntroUNET works very well on both of these two extremely different scenarios. Nonetheless, we have now shared some guidelines for making this decision: we feel that simply choosing which population to order first based on nucleotide diversity will be sufficient. We state this in the Method when seriation is introduced, as suggested by the reviewer.

Another issue that needs to be addressed is the effect of model misspecification. Although ML methods are in principle assumption-free, the training using simulated data necessarily introduces specific assumptions about the evolutionary scenario being considered. It is unclear, for example, how can training a model using data simulated under a bidirectional introgression when the real scenario consists in unidirectional introgression (or the opposite situation) affect the reliability of the results. It may be that for very well studied introgression cases, it is possible to choose the right scenario to use in the simulations, but this is not true in general. Is the proposed method really of general applicability? Either the authors need to prove that this is indeed the case or make it very clear that their method can only be applied to cases for which there is enough detailed information to make this type of decisions. The Drosophila example represents such a case.

Our response: As discussed above, we have added a new section to the manuscript that shows that IntroUNET is able to accurately detect introgression in the presence of numerous types of model misspecification. Regarding the application of the bidirectional version of IntroUNET to a unidirectional case, we show that this results in hardly any loss in accuracy (Figure S5). As we state in the misspecification section of the Results, “accuracy decreases from ~96% when the correct unidirectional network is used to ~95% when the bidirectional network is used.”

2 Specific comments

1. Description of simulated scenarios: the authors should provide more details on how they simulated the bidirectional introgression scenario. Table 1 provides the distribution for the migration probability but how was this really applied? Did the authors made independent draws for each population or did they use the same value for both? In other words, was the introgression bidirectional and symmetric or bidirectional but asymmetric? If symmetric, it would be important to consider an asymmetric case.

Our response: As noted in our responses above, these were independent draws.

2. Figure 3, panel A: both populations have introgressed alleles but the difference in the degree of introgression between the two populations is enormous. This highlight the need to better explain how the bidirectional migration scenario was implemented.

Our response: We apologize for this confusion, and this has been clarified as noted above.

3. lines 254-257: You should present a figure describing this scenario in supplementary information.

Our response: We have added this figure (Figure S11).

4. lines 309-310: You need to provide a description of the simulation model in supplementary information. Readers should not need to search for another publication to find these details.

Our response: We have included the missing parameters in this section (the mutation and recombination rates), making our description of the simulation model complete. We also now provide a complete description of each parameter listed in Table 2, and link to code that can perform these simulations using the values in this table as input.

5. Figure 4 - ”Same as (A), but for introgression from population 2 to population 1”: Clarify, did you tested on data with introgression in the same direction (from 2 to 1)?

Our response: For panel A, the network was trained and tested on introgression from population 1 to 2, and for panel B the network was trained and tested on introgression from population 2 to population 1. This has been clarified in the legend.

As stated above in response to reviewer 2, this was a sanity check to ensure that two versions of IntroUNET trained on essentially the same problem, but on different channels of the image, yielded the same accuracy.

6. Lines 429-430 and 550-551: this brings about the question of whether this method is of wide

applicability. What happens if you have no previous information about which regions of the

genome are affected by introgression?

Our response: This is an excellent point shared by other reviewers. As discussed in those responses above, to make it easier to apply IntroUNET in cases where testing reveals that accuracy is acceptable only in regions where introgression is present, we have incorporated a window-based classifier to identify introgressed regions prior to identifying introgressed haplotypes. Incorporating this tool into IntroUNET’s repository streamlines this process for users wishing to run our tool.
