# Supplementary material for: IntroUNET: Identifying introgressed alleles via semantic segmentation
Source: PLoS Genet. 2024 Feb 20;20(2):e1010657. doi: 10.1371/journal.pgen.1010657 (PMC10906877; doi:10.1371/journal.pgen.1010657)
Supplement: Attachment — Submitted filename: IntroUNET_responses2.docx [file pgen.1010657.s024.docx]

Our responses to the reviewers are found below. We have also included a pdf with tracked changes.

**Reviewer #1**: I would like to thank the authors for their thoughtful consideration of the reviewers’ comments and their extensive efforts to address all of our concerns. This was a lot of extra work, but well worth it! The improvements to the text have made the technical aspects of the project more accessible, and the additional testing of the method have provided meaningful new insight. In particular, I was really surprised to see an appreciable impact of background selection.

The revised manuscript is good and I do not have any further comments or concerns.

Best regards,
Derek Setter

**Reviewer #2:** The authors have made substantial efforts to address all of the major concerns raised by the reviewers. It is rare enough to merit mention, and I would like to thank the authors for their revisions. The updated version of the manuscript includes many new analyses and results to demonstrate the robustness of the method to various model misspecifications. They also extended the IntroUNET package to improve its accuracy with real genomic data (adding a first step of window-based classification) and they show its applicability to unphased data. Finally, the authors rendered the methods more accessible to non-experts and provided guidelines, which is an important aspect for the readership of Plos Genetics. A minor negative point is that the paper is quite long with many technical details in the main text. Overall, this work is of high quality and I expect that IntroUNET will be very useful to the community for genomic studies on introgression. Therefore, I fully support its publication.

We thank Reviewers 1 and 2 for their kind words, and for their detailed comments to our initial submission that allowed us to produce a better manuscript.

**Reviewer #3:** This is a revised version of a manuscript I reviewed some time ago. My review included three main comments. The first one was focused on the preprocessing steps and how the choice of metrics/algorithms could influence performance of the method. The authors have now explored different distance metrics for the seriation step of the first population and identified those that work best. For the second step, they argue that the choice of the population for the initial step is not a major issue. I agree that with two populations, this may be the case, but it may be very different when more than two populations are considered. Nevertheless, the authors implementation considers a two-population scenario so this is not a major issue. However, the authors should not suggest that their method can easily be generalised to scenarios with more than two populations (c.f., ln. 147).

The second major comment was about the effects of model misspecification, which the authors have addressed in great detail in the present version. Finally, there was also an issue about the applicability of the method, because there was a substantial number of false positives when analysing regions that did not have introgression. To avoid this problem it was necessary to know in advance which regions were introgressed. To solve this issue, the authors now include a windows-based step that allows the identification of regions with introgression.

I only have two relatively minor comments that I would like the authors to address. They all concern the lack of a serious hyperparameter optimisation for the architecture used by the authors. In general, the optimisation step is considered as an important aspect of developing DNNs. Although the authors do acknowledge the absence of this step, they should not make statements that are not properly supported. This is particular the case for the explanation of label smoothing. It is unclear if the value of alpha chosen is high enough to have a regularisation effect. Typically, you need to do some experimentation in order to find the optimal value, but this was not done. Therefore, you cannot really state “We found this extra regularization was helpful for training". You need to present some evidence that you have really checked if it helps to avoid overfitting.

We thank the Reviewer for their continued efforts in evaluating our manuscript. We have addressed the remaining comments as follows:

1) Regarding the extension of the method to more than two populations: it was not our intention to imply that our method would work as well for more than 2 populations as it does for our 2-population tests, but simply that our framework is flexible enough that it could be adapted for higher-dimensional input tensors. We have clarified this point, and now state the following in the Methods:

“Note that *l* = 2 in all experiments in this paper; one could in principle adapt this approach to examine *l* > 2 populations by simply adding additional input channels, but we have not evaluated whether acceptable accuracy can be obtained with higher values of *l*.”

2) Regarding label smoothing, we now include results from an analysis demonstrating that the label smoothing parameter value that we used (alpha=0.01) allows the method to generalize better (as measured by accuracy on the validation set), than both smaller or larger values of alpha (0.001 and 0.1, respectively). This finding is detailed in the fourth paragraph of “Network architecture, training, and evaluation”, the Methods subsection where the label smoothing concept is first introduced, and are shown in Figure S2. Note that alpha species the strength of label smoothing, and that our best performance is obtained at the intermediate value of alpha rather than the smallest value considered. This demonstrates that the inclusion of label smoothing does indeed improve our method’s accuracy.
